# Wearable technology to characterize and treat mild traumatic brain injury subtypes: Study protocol for a randomized controlled trial on biofeedback-based precision rehabilitation (SuBTyPE)

**DOI:** 10.1371/journal.pone.0340867

**Published:** 2026-01-30

**Authors:** Jae W. Lee, Prokopios Antonellis, Peter C. Fino, Robert J. Peterka, Jennifer L. Wilhelm, Kathleen T. Scanlan, Margaret E. Stojak, Jennifer L. Brodsky, Cecilia Monoli, Angela R. Weston, William Liu, Kody R. Campbell, Kelsi Schiltz, Irene Robinson, Christina M. Geisler, Siting Chen, Margaret M. Weightman, Carrie W. Hoppes, Leland Dibble, James C. Chesnutt, Laurie A. King

**Affiliations:** 1 Department of Neurology, Oregon Health & Science University, Portland, Oregon, United States of America; 2 Department of Health and Kinesiology, University of Utah, Salt Lake City, Utah, United States of America; 3 National Center for Rehabilitative Auditory Research (NCRAR), VA Portland Health Care System, Portland, Oregon, United States of America; 4 Army-Baylor University Doctoral Program in Physical Therapy, Joint Base San Antonio, Fort Sam Houston, San Antonio, Texas, United States of America; 5 School of Public Health, Oregon Health & Science University, Portland, Oregon, United States of America; 6 Courage Kenny Research Center-Allina Health, Minneapolis, Minnesota, United States of America; 7 Joint Base San Antonio-Lackland Air Force Base, San Antonio, Texas, United States of America; 8 Department of Physical Therapy & Athletic Training, University of Utah, Salt Lake City, Utah, United States of America; 9 Department of Family Medicine, Oregon Health & Science University, Portland, Oregon, United States of America; PLOS: Public Library of Science, UNITED KINGDOM OF GREAT BRITAIN AND NORTHERN IRELAND

## Abstract

**Background:**

Rehabilitation of persistent imbalance in people with mild traumatic brain injury (mTBI) is challenging, and responsiveness to rehabilitation is often suboptimal. One reason for suboptimal outcomes may be patient heterogeneity within rehabilitation referrals. Specifically, people with greater vestibular and/or ocular-motor (V/O) symptoms may respond better to vestibular rehabilitation therapy (VRT) compared to those with greater mood or cognition symptoms. Poor performance of exercises may also explain suboptimal outcomes. This study aims to 1) assess if a wearable sensor-based multidimensional biofeedback system could enhance rehabilitation, 2) examine responsiveness to rehabilitation depending on the severity of V/O deficits, 3) characterize the impact of V/O deficits on gait and turning during seven days of unsupervised daily living and establish normative mobility data from active-duty service members.

**Methods:**

This study is a single-blinded randomized controlled trial involving 100 individuals experiencing persistent symptoms from subacute and chronic mTBI. Participants will be randomized into VRT with or without sensor-based biofeedback. Both groups will receive a 6-week VRT. All participants will be tested for balance, gait, turning, and V/O performance before and after VRT. We will compare the efficacy of VRT with or without biofeedback, stratified by the severity of V/O symptoms. Additionally, a subset of 50 participants with mTBI and 40 healthy active-duty service members will wear inertial sensors for seven days to quantify daily mobility. We will use the data to examine if the severity of V/O deficits following mTBI impacts daily mobility and to establish normative data for daily living mobility from military service members.

**Discussion:**

This study will be the first clinical trial to investigate whether wearable technology can improve rehabilitation outcomes for those with V/O symptoms by providing real-time biofeedback during rehabilitation. This work will also help to identify individuals with sensorimotor deficits associated with V/O subtypes. These results will enhance the assessment and rehabilitative care following mTBI by integrating objective measures to identify and address V/O subtypes. Furthermore, establishing normative data for daily living mobility from service members will aid in return-to-duty decision making following mTBI.

**Trial registration:**

This protocol is registered on ClinicalTrials.gov under the number NCT06381674. Registered on April 04, 2024. Recruited period from June 2024 to September 2028. https://clinicaltrials.gov/study/NCT06381674. Trial Protocol v1 (Dated November 14, 2023)

## Introduction

Each year in the United States, approximately 1.7 million individuals experience a traumatic brain injury (TBI), with approximately 80% categorized as mild TBI (mTBI) [1–4]. Complaints of impaired balance are common after mTBI [5,6] and ongoing balance difficulties contribute significantly to anxiety, and present challenges in returning to work, sports, or military duty [7]. The societal cost of TBI, including lost productivity, was estimated at $60.4 billion annually [8,9]. Persisting balance deficits may explain the observed threefold increase in the risk of subsequent mTBI and heightened susceptibility to musculoskeletal injuries among athletes and soldiers with a recent mTBI history [10–14]. Similarly, the rate of lower extremity injury among active-duty service members after an mTBI was 38% greater than healthy service members [12].

While vestibular rehabilitation is recommended for people with persisting deficits following mTBI, rates of responsiveness to rehabilitation are often suboptimal [15,16]. Recent systematic reviews that examined the effects of rehabilitation after mTBI concluded that most studies were small and lacked standardization, though there was weak yet promising evidence supporting vestibular rehabilitation [15,16]. Another systematic review indicated that subthreshold aerobic exercise, as well as cervical, vestibular and/or ocular-motor (V/O) therapies might alleviate symptoms but not reduce recovery time and that V/O exercises showed limited evidence for improvements in symptom scores [17]. There is a need for a larger, well-designed study to determine the efficacy of vestibular rehabilitation for those with mTBI, specifically individuals with V/O symptoms.

One explanation for suboptimal outcomes in vestibular rehabilitation could stem from inadequate performance of rehabilitation exercises by the patients. Effective vestibular rehabilitation typically involves focus on tasks that prioritize gaze and postural stability during dynamic movements. Gaze stability exercises typically involve gradually increasing the speed and range of head motions while maintaining visual focus on targets. Postural stability exercises typically involve gradual increase in the range of head and body motions while maintaining a stable center of gravity during static and dynamic balance exercises [18]. Recent works showed that individuals with mTBI exhibited reduced head motion, despite normal gait speed, during walking with head turns [19,20], which has important implications for a successful return-to-activity, duty, and sport [19]. Adhering to principles of motor learning, providing feedback on performance is crucial for effective rehabilitation [21–23]. However, quantifying and delivering feedback during balance rehabilitation poses difficulties due to the subtlety of movements (i.e., increased postural sway or slowed head turns) that are often not visually discernible. Current biofeedback methods often fall short in providing feedback on complex multidimensional movements, such as walking with head turns [24].

Another contributing factor to the suboptimal response rates to rehabilitation following mTBI could be the lack of targeted rehabilitation. Recent research has categorized mTBI subtypes based on common clinical presentations that include cognitive, ocular-motor, headache/migraine, vestibular, and anxiety/mood; with cervical strain and sleep disturbance as associated conditions that can occur across subtypes [25–27]. Correctly identifying the prominent subtype may be crucial for determining the most appropriate rehabilitative interventions. However, characterization of mTBI subtypes can be challenging partly due to overlapping and equally prominent subtypes [26]. While V/O subtypes are most likely to benefit from vestibular rehabilitation, V/O symptoms are often under-represented in subjective rating scales [28]. This study aims to refine the identification of V/O subtypes via instrumented V/O functions and to ultimately enhance rehabilitation outcomes.

Finally, another consideration in appropriately identifying V/O subtypes is the snapshot nature of clinical assessments, which may not reflect V/O symptoms experienced in one’s daily life. For example, turning in daily life requires rapid sensorimotor reweighting and ocular-motor reflexes to reorient the eyes, head, trunk, and pelvis towards the new travel direction, and might differ from turning in standardized laboratory settings. Individuals with prominent V/O symptoms post-mTBI might navigate their environment differently to avoid symptom exacerbation. Wearable sensors allow for the collection of quantity and quality of gait and turning during daily life mobility [29,30], and previous research reported impaired quality of movement during daily life in people with chronic mTBI [31,32]. To date however, there have been no studies that examined the relationship between the severity of V/O symptoms with daily life mobility. Additionally, as active-duty military service members have shown to have higher physical activity levels than civilians [33], an assessment of gait and turning during daily life mobility might be more insightful for evaluating readiness for return-to-duty than common clinical assessments. To date, normative gait and turning data for active-duty military service members during daily life mobility has not been established.

This study has three aims: 1) determine if multidimensional real-time biofeedback using novel wearable technology, which simultaneously measures head/trunk movement and postural sway during standing and walking rehabilitative exercises, improves outcomes after vestibular rehabilitation compared to standard care; 2) investigate the responsiveness of rehabilitation based on the level of V/O symptom severity and the strength of the relationship between patient-reported and clinical/instrumented assessments of V/O measures; and 3) determine the differential impact on daily life mobility (quality of gait and turning) in individuals with moderate to severe V/O symptoms compared to those with minimal V/O symptoms. We will also calculate healthy normative data for daily life mobility measures in active-duty military service members. We hypothesize that augmenting rehabilitation with wearable sensors to measure and provide feedback will improve rehabilitation outcomes. Secondly, we hypothesize that people with moderate to severe V/O symptoms (V/O HI) will demonstrate a higher rate of responsiveness to rehabilitation compared to those with minimal V/O symptoms (V/O LO). We also hypothesize that there will be small to moderate relationships between patient-reported and clinical/instrumented assessments that relate to V/O measures. Our third hypothesis is that gait and turning quality, as measured by wearable sensors during 7 days of daily life, will be worse in people with V/O HI than those with V/O LO.

## Methods

The three aims of this study are detailed in Table 1.

**Table 1:** Overview of study aims and milestones.

Aim	Focus	Protocol	Participants	Milestones
I	Multidimensional real-time bio-feedback using wearable sensors during rehabilitation	Aim I: Randomized into biofeedback or standard care	100 mTBI subjects: OHSU (n = 50) UU (n = 50)	Determine if multi-dimensional real-time biofeedback improves efficacy of rehabilitation Summarize qualitative assessments from the treating physical therapist to help inform next steps for clinical implementation
II	Responsiveness to rehabilitation and V/O subtype classification	Aim II: Calculate percentage of responders to rehabilitation across V/O subtypes; explore correlations between types of measures used for V/O subtype classification	All 100 mTBI subjects from Aim I	Calculate responsiveness to rehabilitation across severity of V/O deficits. Explore the strength of correlation between subjective and objective measures of V/O function
III	Continuous monitoring of daily-life function with wearable sensors	Aim III: Wore instrumented socks for ~7 days	50 mTBI subjects: OHSU (n = 25) UU (n = 25) 40 healthy active duty service members FSH (n = 40)	Characterize daily-life mobility based on V/O subtypes. Calculate healthy normative daily life data for active-duty service members

mTBI: mild traumatic brain injury; OHSU: Oregon Health & Science University; UU: University of Utah; FSH: Joint Base San Antonio – Fort Sam Houston; V/O: Vestibular and/or ocular-motor;

### Trial design

**Fig 1:**
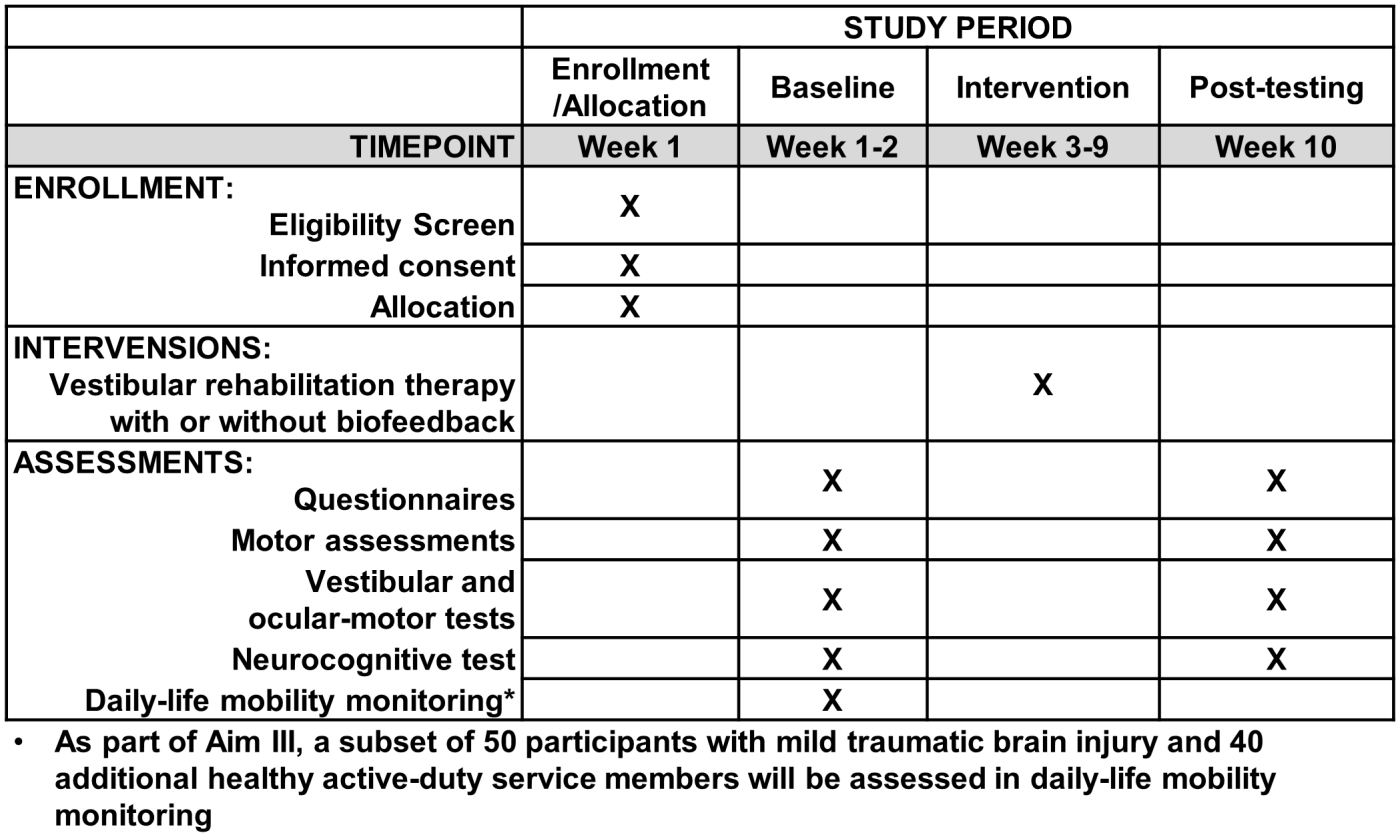
Participant timeline: Schedule of enrollment, interventions, and assessments.

**Fig 2:**
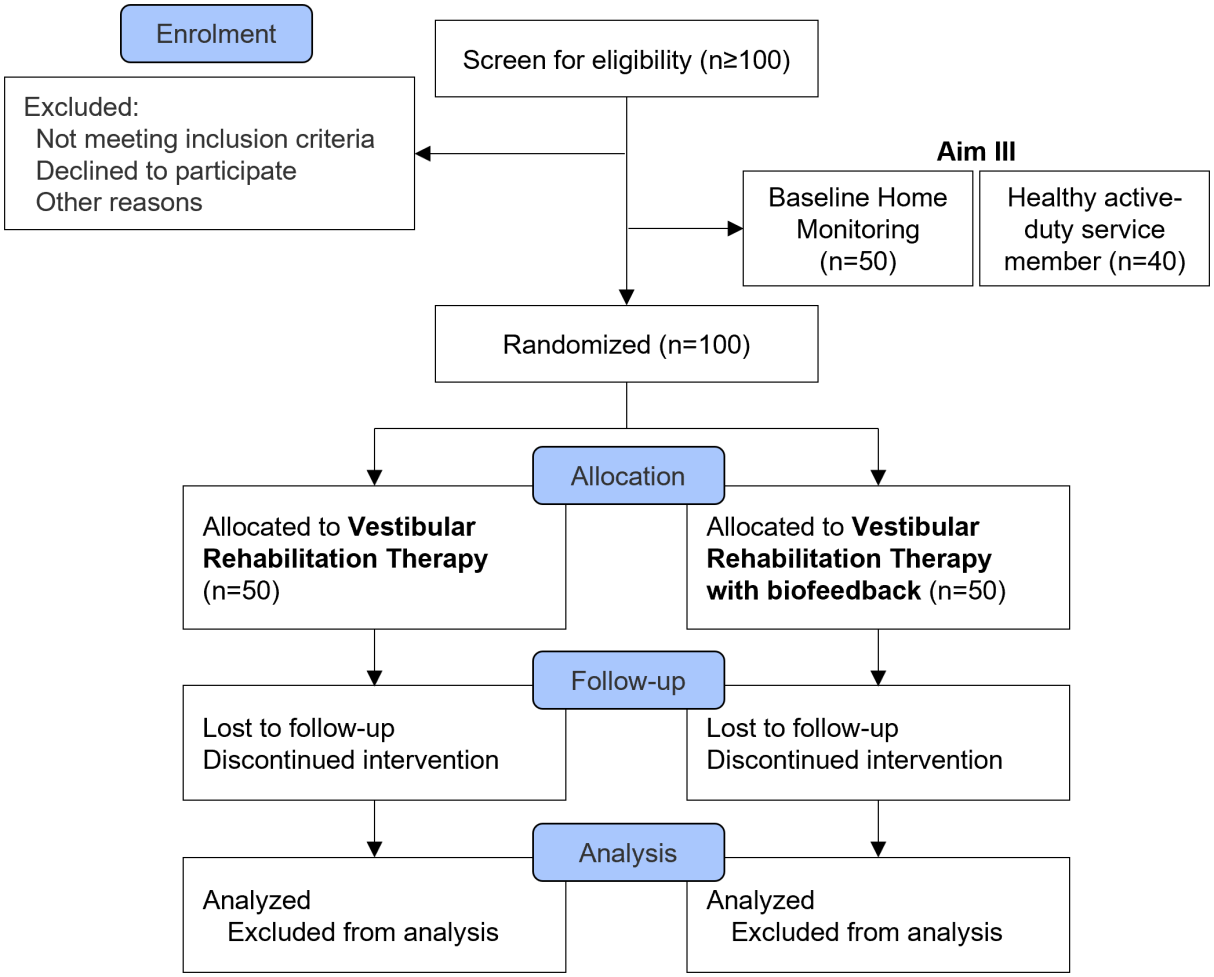
Study design.

This protocol is a single-blinded, randomized controlled trial. The study received approval from a joint Oregon Health & Science University and Veterans Affairs Portland Health Care System Institutional Review Board (eIRB #25890) and will be executed in compliance with the Declaration of Helsinki. The trial is registered on ClinicalTrials.gov (Identifier: NCT06381674; date of registration: 2024-04-04). This protocol adheres to the Standard Protocol Items: Recommendations for Interventional Trials schedule (Fig 1). The study design is illustrated in Fig 2.

### Participants and eligibility criteria

This study will involve 100 participants experiencing persistent symptoms from mTBI and 40 healthy controls that are active-duty service members. Participants will be recruited between June 2024 to January 2027. The data collection will be expected to be completed by April 2027, and the results are expected January 2028. Testing and rehabilitation (Aim I & II) for civilians will be conducted at Oregon Health & Science University (OHSU), Veterans Affairs (VA) Portland Health Care System, and University of Utah (UU). Home monitoring with wearable sensors (Aim III) will include a subset of participants at OHSU and UU as well as active-duty military service members at Joint Base San Antonio- Fort Sam Houston (FSH), in San Antonio, Texas.

Recruited participants must: 1) have been diagnosed with mTBI according to VA and Department of Defense criteria [34,35], 2) be 18−60 years old, 3) be capable of standing unassisted for at least 10 minutes, 4) be outside of the acute stage (> 2 weeks post-mTBI) but within 6 months of their most recent mTBI and still reporting symptoms, 5) demonstrate some level of measurable V/O deficits based on the Concussion Clinical Profile Screening (details provided below), 6) have sufficient vision (corrected or uncorrected) for unassisted reading and performance of daily personal tasks and independent community ambulation, and 7) have adequate hearing (without amplification) for engaging in close-range personal or telephone conversations. For Aim III, active-duty military service members must have no recent history (< 3 years) of mTBI and no residual symptoms from any previous mTBI.

Exclusion criteria for all aims include participants that: 1) have a current or past history of any other injury, medical, or neurological illness that could potentially explain balance or vision deficits (e.g., central nervous system disease, stroke, epilepsy, greater than mild TBI, Meniere’s disease, bilateral vestibular loss, recent lower extremity or spine orthopedic injury requiring activity modifications (or duty limitations for service members)), 2) meet criteria for moderate to severe substance-use disorder within the past month as outlined by Diagnostic and Statistical Manual of Mental Disorders-V, 3) display behavior that would significantly interfere with validity of data collection or safety during the study, 4) are in significant pain during evaluation (> 7/10 by patient subjective report), 5) are pregnant (due to balance considerations), 6) have been hospitalized for any brain injuries (separate from emergency department visits), 7) have significant joint pain or recent musculoskeletal injury that limits walking or mobility, 8) have had any major surgeries in the past year or amputation, 9) use an assistive device, 10) are unable to stand barefoot, 11) currently are in or have received rehabilitation services or physical therapy for symptoms related to their mTBI within 1 month of the screening date.

#### Justification of V/O symptom classification.

The concept of individuals with mTBI having one primary subtype was developed on people in the acute stages of mTBI [27]. In contrast, those with sub-acute or chronic mTBI often develop a more heterogeneous profile [36]. Since recruited participants are in the sub-acute (i.e., 14–90 days) to the chronic phases (i.e., 90–180 days) of mTBI, this study proposes an alternative approach to categorize individuals with V/O subtypes, based on clinical measures with literature-based cutoffs based on current literature and prior project data (Grant No. W81XWH-18-2-0049 and W81XWH-17-1-0424). Clinical subjective measures include Concussion Clinical Profile Screen (CP Screen) [37], Dizziness Handicap Inventory (DHI) [38], and Vestibular/Ocular Motor Screening (VOMS) [39]. Participants will be classed into two groups: 1) V/O LO if V/O subscores of CP Screen are between 0.61 and 1.5 [40], DHI is less than 54 points [38], and VOMS total score is less than or equal to 49 points (Grant No. W81XWH-18-2-0049 and W81XWH-17-1-0424) [41], 2) V/O HI if 2 of the following criteria are met: V/O subscores of CP Screen are greater than or equal to 1.6 [40], DHI is greater than or equal to 54 points [38], or VOMS total score is greater than or equal to 50 points (Grant No. W81XWH-18-2-0049 and W81XWH-17-1-0424) [41]. Those with V/O subscores from the CP Screen that are less than 0.6 will be excluded from the study (Minnesota SCI & TBI Research Grant: 128519).

### Recruitment, randomization, and allocation

Individuals with mTBI will primarily be recruited from the OHSU Concussion Clinic, OHSU Hospital System, the UU Concussion and Brain Injury Program, and the UU Health System. Active-duty military service members will be recruited from FSH, primarily from personnel at the U.S. Army Medical Center of Excellence. Recruitment efforts will include word-of-mouth referrals, distribution of flyers and posters within these facilities, and briefings for assigned personnel.

Participants with mTBI will be randomized into multimodal rehabilitation intervention consisting of either 1) vestibular rehabilitation therapy or 2) vestibular rehabilitation therapy with augmented real-time biofeedback using wearable sensors. Following the baseline assessments, participants will be randomized with an automated Research Electronic Data Capture system database algorithm that is site controlled and includes phase of recovery based on time since injury and V/O HI or LO classification at baseline. The allocation sequence is concealed until participants are enrolled and assigned to an intervention. Given the significance of V/O symptoms in our hypotheses, we will employ a stratified block randomization method. This approach will stratify based on V/O HI and V/O LO to ensure that both rehabilitation interventions receive an approximately equal number of participants with similar severity of V/O symptoms. Block randomization will assist in ensuring balanced allocation to the two intervention groups, with block sizes (2, 4, 6, or 8) varied to mitigate potential selection bias.

### Blinding

Participants will remain unaware of the favored intervention. The researchers delivering the intervention will be aware of participants’ assigned intervention. The researchers conducting the pre- and post-tests and analyzing the data will be unaware of the group assignments, ensuring blinding throughout the study.

### Assessment procedures

The pre-screening for inclusion and exclusion criteria will be conducted via phone. All eligible participants with mTBI will come into either OHSU or UU for the informed consent process. An investigator will verbally explain the consent forms to the participants and allow ample time to read through the consent forms (S2). Participants will confirm their consent to participate by signing the consent forms.

Two days of baseline testing (approximately six total hours) will occur in the Balance Disorder Laboratory at OHSU, National Center for Rehabilitative Auditory Research at VA Portland Health Care System, Neuromechanics and Applied Locomotion Laboratory, and Balance and Mobility Clinic at UU. Participants will complete validated questionnaires; motor assessments of standing and reactive balance, gait, and turning; standard vestibular and ocular-motor tests; and a computerized neurocognitive test (see Tables 2 and 3 for detailed lists of tests). Following baseline assessments, a convenience sample of 50 individuals with mTBI, with approximately equal number of participants with V/O HI and LO, will undergo 7 days of daily-life mobility monitoring. Subsequently, participants will receive a 6-week intervention, detailed below. Post-testing will be conducted after completing the intervention sessions. Additionally, 40 active-duty service members will undergo 7 days of daily-life mobility monitoring at FSH. The overview of the study is shown in Fig 2.

**Table 2:** Patient-reported subjective questionnaires.

Domain	Test	Purpose
General	Patient Global Impression of Change (PGIC) [42–44]	Perceived change
	Neurobehavioral Symptom Inventory (NSI) [45]	Symptoms
	Quality-of-Life after Brain Injury (QOLIBRI) Scale [45]	Quality-of-life
	International Physical Activity Questionnaire (IPAQ) – Short [46,47]	Physical activity
	Patient-Reported Outcomes Measurement Information System (PROMIS) Short Form 4a [48]	Satisfaction with social roles
mTBI Subtypes	Concussion Clinical Profile (CP) Screen [37]	Concussion symptoms
	Dizziness Handicap Inventory (DHI) [38]	Vestibular/ Ocular
	Vertigo Symptom Scale (VSS) [49]	Vestibular
	Convergence Insufficiency Symptom Survey (CISS) [50]	Ocular
	Headache Impact Test (HIT) – 6 [51]	Headache
	Hospital Anxiety & Depression Scale (HADS) [52]	Anxiety/ Mood
mTBI-associated conditions	Neck Disability Index (NDI) [53]	Cervical strain
	Insomnia Severity Index (ISI) [54]	Sleep

**Table 3:** Clinical and instrumented assessments.

Domain	Test	Purpose
Standing Balance	Modified Balance Error Scoring System (mBESS)^1,2^ [55]	Instability in standing conditions
	Modified Clinical Test of Sensory Interaction in Balance (mCTSIB)^1,2^ [56]	Sensory contribution to balance
	Central Sensorimotor Integration (CSMI) test^2*^ [57–59]	Identification of balance control system
Reactive Balance	Reactive stepping^1,2^ [60–62]	Balance recovery with stepping
Gait and turning	Functional Gait Assessment (FGA)^1,2^ [63]	Gait-related activities
	4-Item Hybrid Assessment of Mobility for mTBI (HAM-4-mTBI)^1^ [64]	Gait-related activities
	1-min walk (single and dual task)^1,2^ [65]	Straight path walking with 180^o^ turns
	Tandem gait (single and dual task)^1,2^ [66,67]	Straight path tandem walking with 180^o^ turns
	Complex Turning Course (CTC)^1,2^ [68]	Path with 45^o^, 90^o^, 135^o^ turns
Vestibular and Ocular-motor	Vestibular Ocular Motor Screening (VOMS)^1^ [39]	Assessment of V/O systems
	Clinical Dynamic Visual Acuity (DVA)^1^ [69]	Visual acuity during head motion
	Video Head Impulse Test (vHIT)^2^ [70]	Vestibular-ocular reflex assessment
	Smooth pursuit^2^ [71–73]	Ocular-motor assessment
	Random saccades^2^ [73]	Ocular-motor assessment
	Antisaccasdes^2^ [73]	Ocular-motor assessment
Neuro-cognition	Automated Neuropsychological Assessment Metrics (ANAM)^1^ [74]	Computerized cognitive assessment

Covariates: Age, sex, previous mTBI history, Post-Traumatic Stress Disorder checklist

^1^ Clinical tests, ^2^Instrumented tests

* performed only at Oregon Health & Science University

#### Primary outcome measures.

The primary outcome measure will be the Patient Global Impression of Change (PGIC) [42–44], which will be collected during the post-testing as part of the validated questionnaires. The PGIC was selected by Department of Defense stakeholders from the Office of Outcomes and Assessment at the Defense and Veterans Brain Injury Center in 2014 as one of the core measures for assessing mTBI healthcare outcomes regarding treatment benefits [42–44].

#### Secondary outcome measures.

Secondary outcome measures will be derived from questionnaires, motor assessments, vestibular and ocular-motor tests, and a neurocognitive test (Table 3). Many of these measures adhere to the Common Data Elements outlined by the National Institute of Neurological Disorders and Stroke Common Data Elements TBI Project [75]. Additional details regarding data collection and analysis procedures are summarized below.

Instrumented evaluations of standing and dynamic balance, gait, and turning will utilize synchronized inertial sensors (APDM Wearable Technologies, a Clario company, USA). These sensors are equipped with 3D accelerometers (range ± 6 g), gyroscopes (range ± 2000°/s), and magnetometers (range ± 6 gauss). Sensors will be affixed to the head, sternum, lumbar spine, bilateral wrists, and bilateral feet using elastic Velcro bands. Data from the inertial sensors, collected at a rate of 128 Hz, will be synchronized and wirelessly transmitted to a laptop for automatic generation of metrics, while the raw data will be retained for further analysis.

Participants at OHSU will also undergo a Central Sensorimotor Integration (CSMI) test while standing on the NeuroCom platform (SMART Equitest CRS, Natus Medical Inc., USA) [57–59]. During the CSMI test, custom surface and/or visual perturbation will be applied while the participants are tasked with maintaining balance. Applied perturbations and corresponding body sway responses will be used to quantify balance control characteristics, including sensory weights, time delay, and motor activation parameters that determine corrective ankle torque generation [58].

Vestibular function assessment will involve video head impulse tests (vHIT) (Otometrics ICS Impulse and Otometrics ICS Chartr 200, Natus Medical Inc., USA) [70], to detect any reductions or asymmetries in vestibular ocular reflex function. Ocular-motor function evaluations (VisualEyes 525, Interacoustics, Denmark; I-Portal Neuro Otologic Test Center, Spryson America Inc., USA) will include 1) horizontal (0.3 and 0.7 Hz) and vertical (0.3 Hz) smooth pursuit tests measuring eye movements tracking sinusoidal target motion, 2) horizontal and vertical saccade tests measuring eye motion characteristics when tracking visual targets appearing in pseudo-random locations, and 3) horizontal antisaccade test measuring eye movements when subjects are requested to perform a saccade in the opposite direction to a visual target occurring in a random direction. Recorded eye movements are analyzed to provide quantitative metrics such as vHIT vestibular-ocular reflex gain (comparing eye velocity to head velocity), vHIT gain asymmetry, smooth pursuit eye velocity gains and asymmetry, saccade onset latency and initial accuracy, and antisaccade directional errors.

### Daily-life mobility monitoring

**Fig 3:**
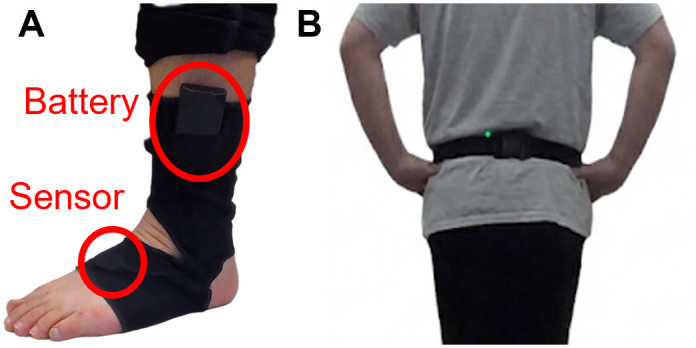
Instrumented socks with inertial sensors. **(A)** Inertial sensors placed on the top of foot and battery on the side **(B)** Inertial sensors on the posterior side of the waist, near the lumbar region. The individual shown in Fig 3 has given written informed consent (as outlined in PLOS consent form) to publish these images.

Participants will utilize Opal Instrumented Socks (APDM Wearable Technologies, a Clario company, USA), consisting of a thin elastic fabric embedded with inertial sensors, to monitor their mobility in daily activities (Fig 3). The sensors will be positioned on the dorsum of the foot with the battery situated above the lateral malleolus [30,31]. Additionally, participants will wear an Opal sensor on a belt, positioned over the lumbar area. These sensors will be worn throughout the day for approximately 8–10 hours over the course of 7 days, with nightly charging. At the end of the 7-day period, participants will return the sensors and the journal, which records the times that the sensors were put on and remove, either by mail or in-person. The quality and quantity of turns measured by wearable sensors will be calculated [29–31].

### Intervention

The intervention will consist of twelve sessions, each lasting approximately 60 minutes, delivered twice per week for 6 weeks. The intervention will be administered and/or supervised by a licensed physical therapist. The physical therapy program is based on the Clinical Practice Guideline recommendations [2,76], which advocate for a multimodal physical therapy approach tailored to the participant’s impairments and symptoms and progressed to their tolerance. During the first rehabilitation session, participants will be screened for benign paroxysmal positional vertigo with Dix-Hallpike and supine roll tests, and positive findings will be treated according to the benign paroxysmal positional vertigo Clinical Practice Guideline recommendations [77]. Additionally, participants will be assessed for cervical dysfunction with the Numeric Pain Rating Scale, Active Range of Motion (ROM) testing, Head-Neck Differentiation Test [78], and the Cervical Flexion Rotation Test [79]. If cervical dysfunction is present, it will be treated with a home exercise program. Our intervention includes six exercise categories: ocular-motor, standing balance, perturbations, gaze stabilization, visual motion sensitivity, and dynamic balance related to walking tasks (Table 4). Exercises will be advanced in each category through changes in surface conditions (i.e., firm, foam, Both Sides Utilized (BOSU) [BOSU® Fitness, LLC, USA], or rocker board), visual conditions (i.e., blank background, high contrast checkerboard, or moving lights), and the addition of cognitive or motor dual-tasking (Table 4) [80].

**Table 4:** Multimodal rehabilitation sessions.

Domain	Areas of Focus	Description						
Cervical	1. Stretching 2. Strengthening	Exercises will be provided if the participant is positive for the cervical modifier on the Concussion Clinical Profile Screening performed at baseline testing (≥2) or if the physical therapist deems necessary from clinical judgement. The goal is to prevent the cervical spine from limiting range of motion (ROM)/ velocity during vestibular exercises.						
Benign Paroxysmal Positional Vertigo	1. Posterior canal 2. Horizontal canal	Posterior and horizontal canals will be assessed on the first day of rehabilitation; if the participant tests positive, either Epley maneuver or Barbeque Roll will be performed until canal is clear.						
**Domain**	**Areas of Focus**	**Multidimensional** **Biofeedback**	**Challenges**					**Goals**
			Velocity	ROM	Surface	Visual	Dual Task	
Ocular-motor	1. Smooth pursuits 2. Saccades 3. Accommodation	- Cervical ROM/ velocity - Balance stability	x		x	x		Improve ocular-motor control while minimizing head motion and sway
Standing Balance	1. Static balance 2. Head turns*	- Cervical/ trunk ROM/ velocity - Balance stability	x	x	x	x	x	Minimizing sway while maximizing head ROM/ velocity
Perturbation (on unstable surface)	1. Static balance 2. Head turns*	- Cervical/ trunk ROM/ velocity - Balance stability	x	x		x	x	Minimizing sway while maximizing head ROM/ velocity
Gaze Stabilization	1. Vestibular-ocular reflex (VOR) x 1*	- Cervical/ trunk ROM/ velocity - Balance stability	x	x	x	x		Minimizing sway while maximizing head ROM/ velocity
Visual Motion Sensitivity	1. Horizontal 2. Vertical 3. Diagonal	- Cervical/ trunk ROM/ velocity - Balance stability	x	x	x	x		Maximizing head/ sternum ROM/ velocity
Dynamic Balance	1. Walking + head turns* 2. Walking + VOR* 3. Tandem walking + head turns* 4. Lunge + trunk rotation	- Cervical/ trunk ROM/ velocity - Gait stability	x	x	x	x	x	Minimizing trunk sway while maximizing head ROM/ velocity

* Horizontal and vertical directions.

**Fig 4:**
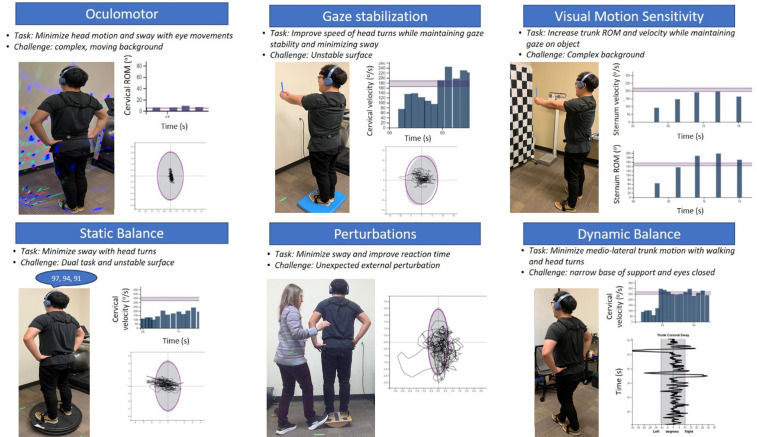
Exercise stations of the multimodal rehabilitation intervention. The individuals shown in Fig 4 have given written informed consent (as outlined in PLOS consent form) to publish these images.

The biofeedback group will receive biofeedback on various parameters including head velocity or ROM, sternum velocity or ROM, and postural sway (Fig 4). Target parameters for each exercise will be based on participants’ performance and previously collected data from healthy controls as target zones. Biofeedback will be provided through audio channels and can be adjusted to provide either continuous or intermittent feedback to maximize motor learning. The standard of care group will complete the same exercise protocol while wearing the sensors but will not receive any biofeedback. For both study groups, surface progression will be standardized so that all participants perform exercises on firm ground for sessions 1–4, foam for sessions 5–8, and BOSU or rocker board for sessions 9–12. If for any reason a participant cannot tolerate the progression, they will remain at the previous level until they can be safely progressed. Visual challenges will be individualized to the participant for each exercise for both groups.

All participants will receive a home exercise program (HEP) consisting of three exercises. These exercises will be selected from five categories: ocular-motor, static balance, gaze stabilization, visual motion sensitivity, and dynamic balance. The selection will include exercises tailored to individual needs and will be advanced based on the participant’s tolerance, including changes in surface condition and visual stimuli. Participants who score ≥2 on the cervical modifier on the pre-rehabilitation intervention CP Screen or those for whom the physical therapist determines that the cervical spine limits their ability to perform the intervention will be educated on an additional cervical exercise component for their HEP. Compliance with the HEP will be tracked through journals collected at each session.

### Community based participatory research

Our study team works with a Community Advisory Board (CAB) including organizational partners, clinicians, and lived experience consultants. This board will ensure a Community-Based Participatory Research approach throughout the planning, design, execution, analysis and dissemination of this research. The input from all members will come from moderated discussions (quarterly) and will be included during the entirety of the study. Currently, the CAB for this study includes physical therapists, physical therapy students, athletic trainers, occupational therapists, Veterans, and lived experience consultants (i.e., individuals who have sustained a mTBI in the past). The inputs from our CAB have guided development of the rehabilitation program, treatment tolerance expectations with the planned rehabilitation approach, symptom education and mitigation strategies, techniques to address barriers to care, and additional topics for this project.

### Safety issues

Any adverse events occurring during either physical therapy sessions or data collection will be documented and reported to the Institutional Review Board.

### Data management and monitoring

Team members will manage data entry tasks, inputting data into both Research Electronic Data Capture system and the OHSU Balance Disorders Laboratory database. Physical copies of records will be securely stored in a locked office cabinet at OHSU and UU. Instrumented de-identified data will be gathered on a laptop computer with password protection and data encryption at OHSU and UU. After completing the protocol, this data will be uploaded to an OHSU secure server, where the Balance Disorders database resides. To ensure accuracy, all data will undergo quality-checking quarterly by an individual not directly involved in the study’s day-to-day operations.

The OHSU Institutional Review Board conducts annual reviews to ensure participant safety and data integrity, including monitoring of adverse events, protocol deviations, and general procedures. Quarterly and annual reports are submitted to the Department of Defense, providing specific updates on study proceedings and progress on the scope of work. All annual reviews are also submitted to Office of Human Research Oversight, to ensure that important protocol modifications are communicated to relevant parties.

### Stopping the trial

The study will be stopped prior to its completion if (1) the intervention is associated with adverse effects that question the safety of the intervention, (2) new information becomes available during the trial that necessitates stopping the trial, or (3) there are other situations that warrant stopping the trial.

### Sample size calculation

Sample size calculations for Aim I and II were based on pilot data on participants with mTBI who received rehabilitation during the development phase of the biofeedback system (Grant No. W81XWH-17-1-0424). A group of 43 participants with mTBI were randomized into standard vestibular rehabilitation therapy or vestibular rehabilitation therapy while using wearable sensors at home (unsupervised). The wearable sensors measured movement during home exercises and that information was discussed with participants during subsequent therapy sessions. For those using wearable sensors, feedback was not provided in real-time but was instead delivered during the subsequent physical therapy session after the sensors had been used during home exercise. Participants rated their perception of change using the PGIC at the end of rehabilitation. In the pilot study, participants who provided sensor data had a mean PGIC score 0.5 ± 1.3 points higher than the standard vestibular rehabilitation group. For Aim I, we expect a larger mean difference (i.e., 0.75) because the sensor group will receive immediate feedback. Also, we anticipate a smaller standard deviation because of more consistent therapeutic results (i.e., SD = 1). We will recruit 50 participants per group. After accounting for an anticipated dropout rate of 25%, we will have 38 participants per group. This sample size could achieve 90% power based on the two-sample t-test to detect the difference of 0.75 ± 1.00 using a two-sided test, alpha at 0.05.

For Aim III, we used pilot data collected from eight participants on objective daily life mobility metrics. Participants were categorized as V/O HI (n = 3) or as V/O LO (n = 5) based on their vestibular testing. From the pilot data, mean differences and common standard deviation between the two groups for turn angle was 4.48 ± 4.14^o^, turn angle variability was 0.018 ± 0.016, and peak turn velocity was 7.95 ± 4.47^o^/s. Additionally, these three metrics had very strong effect sizes (1.1, 1.1, and 1.8, respectively). Therefore, we conservatively determined the sample size calculation with an effect size of 0.90 when comparing two groups. To gain at least 80% power to detect group difference, we need at least 21 subjects for each group (a total of 42 participants), alpha at 0.05. Given an estimated completion rate greater than 85%, we plan to enroll 25 subjects per group (n = 50).

### Statistical analysis

For Aim I, responsiveness to rehabilitation will be determined by the participants’ score on the PGIC after rehabilitation – the primary outcome. We will first conduct a Mann-Whitney U test to assess group differences. Next, PGIC scores will be dichotomized by “responder” or “non-responder”. We will classify participants who score 6 or above on the PGIC as “responders” and those who score 5 or below as “non-responders” [42]. To compare the groups, the Chi-square test will be used to compare responders vs. non-responders in PGIC scores between groups. Then we will conduct a logistic regression on responder vs. non-responder as the binary outcome to assess differences between groups accounting for covariates (e.g., age, sex, Post-Traumatic Stress Disorder, etc.). Additionally, we will conduct ordinal logistic regression on PGIC score levels to examine group differences accounting for covariate effects. Secondary outcomes (e.g., patient-reported symptoms, clinical/instrumented objective assessments) will be measured at baseline and post-rehabilitation, and pre-post change will be calculated for each participant. To compare the differences in pre-post changes in secondary outcomes between the real-time biofeedback group and the standard vestibular rehabilitation group, we will conduct a two-sample t-test or Mann-Whitney U test for each secondary outcome. We will also employ mixed effects models to examine the differences in change in secondary outcomes between the real-time biofeedback and standard vestibular rehabilitation group. Two fixed effects will be included in the model: 1) group effect (real-time biofeedback vs. standard vestibular rehabilitation) and 2) time effect (baseline and post-rehabilitation). The interaction between group and time (group x time) will be included to assess the differences in pre-post change between groups. Random intercepts will be included to account for the clustering effect within subjects over time, and covariates will be included in the fully adjusted model. We will use an inverse probability weighting approach to account for participant attrition in the study and perform sensitivity analyses for missing data [81].

For Aim II, we first will examine the distribution of PGIC scores and the proportion of responders vs. non-responders in V/O HI group and V/O LO group, regardless of randomization group assignment. Then, we will assess the PGIC as an ordinal outcome variable and test group differences (V/O HI vs. V/O LO) in responsiveness using ordinal logistic regression. Additionally, we will conduct a logistic regression on responder vs. non-responder as the binary outcome to assess differences between V/O HI group and V/O LO group. Both regression models will allow us to control for covariates (e.g., age, sex, days since injury, intervention arm) in the models. We will use the data to obtain estimates to inform future studies and power calculations. Finally, to explore the strength of associations between the patient-reported and clinical/instrumented V/O measures, we will measure the associations using Pearson correlation coefficients for continuous variables and Spearman correlation coefficients for ordinal variables.

For Aim III, participants will be grouped according to their level of severity of V/O symptoms (V/O HI or V/O LO symptoms), regardless of intervention group. To test whether daily life mobility measures and quantitative activity measures differ between V/O HI and V/O LO groups, we will test the metrics for group differences using a two-sample t-test or a Mann-Whitney U test. In addition, we will conduct subsequent analyses to compare metrics between the two groups using the multivariate generalized linear modeling approach. The model will be constructed for each metric separately and will be adjusted for covariates. Other quality and quantity metrics will be examined using the same statistical approach. Data from the healthy military normative group will be collected and examined to provide preliminary data for our next studies exploring daily life mobility in those cleared to return-to-duty after mTBI. We will provide both statistical and graphical evaluation for the distributions of turn quality metrics and quantitative activity measures. The descriptive statistics of these metrics will be reported as mean (standard deviation).

## Discussion

This manuscript describes the protocol for a randomized controlled trial aimed at improving the rate of responsiveness to rehabilitation of balance and gait in people with subacute and chronic mTBI. Specifically, we are testing whether our novel intervention, provided to those with V/O symptoms following mTBI, could improve rehabilitation efficacy by incorporating real-time biofeedback during rehabilitation. This work will also help determine whether those with high V/O symptoms show greater responsiveness to rehabilitation than those with low V/O symptoms and characterize the quality of mobility during daily life.

Despite the importance of providing feedback on performance during rehabilitation, most balance feedback, to date, simply tries to decrease postural sway in static conditions or to improve joint motion during gait, without measuring multidimensional movements, like walking with head turns [24,82]. Our study is uniquely designed to combine real-time feedback based on both the lumbar, trunk, or head movements as well as during standing and walking tasks. If the addition of sensor-based biofeedback during rehabilitation results in greater patient perceived improvements compared to standard vestibular rehabilitation, this will support the use of a biofeedback system for successfully augmenting rehabilitation. Such a biofeedback system also has the potential to provide cohesive and targeted rehabilitation by providing feedback on performance to participants as well as physical therapists.

Among mTBI subtypes, it may be that those with V/O symptoms are most likely to benefit from vestibular rehabilitation compared to those with other subtypes, such as cognitive, headache/migraine, and/or anxiety/mood. If we find that people with higher V/O symptoms are more responsive to rehabilitation compared to those with milder V/O symptoms, we will conclude that accurate and standardized classification of mTBI subtypes, especially for those with V/O symptoms, is crucial for successful rehabilitation. Potential barriers for adequate classification might arise from the subjective nature of common assessment tools used to detect sensorimotor impairment and from V/O symptoms often being under-represented in global mTBI symptom rating scales. As such, if there are small to moderate associations between subjective and objective measures of V/O functions, we will conclude that mTBI subtyping should include both subjective and objective measures for assessments prior to referral for vestibular rehabilitation.

Improved mobility in unsupervised settings, such as at home or in daily life, may be especially important to determine the readiness for return-to-activity, sports, and duty following mTBI. Previously, mTBI has been suggested to negatively affect turn quality during daily life compared to healthy controls [31,32]. If we find that turn quality during daily life is different between those with high and low V/O symptoms, we will conclude that measures of turning quality during daily life can also be used as objective measures to better direct rehabilitation and to track recovery. Additionally, quantified gait and turning measures from healthy service members could be used to establish normative data as a decision tool for determining return-to-duty readiness post-mTBI.

## Conclusion

This study protocol aims to address gaps in the suboptimal responsiveness to rehabilitation following mTBI, as sensor-based biofeedback during vestibular rehabilitation has the potential to improve the efficacy of rehabilitation programs, particularly for individuals with prominent V/O symptoms. The findings from this study could provide insights into the therapeutic benefits of incorporating biofeedback into the rehabilitation program and into objectively assessing one’s readiness to return to activity, sports, and/or military duty post-mTBI.

## Supporting information

S1 File SPIRIT 2025 checklist. (DOCX)

S2 File Trial Protocol and Statistical Analysis Plan v1. (DOCX)

## Abbreviations

TBI: Traumatic brain injury
mTBI: Mild traumatic brain injury
V/O: Vestibular and/or ocular-motor
V/O HI: moderate to severe V/O symptoms
V/O LO: minimal V/O symptoms
OHSU: Oregon Health and Science University
VA: Veterans Affairs
UU: University of Utah
FSH: Fort Sam Houston
CP Screen: Concussion Clinical Profile Screen
DHI: Dizziness Handicap Inventory
VOMS: Vestibular/Ocular Motor Screening
PGIC: Patient Global Impression of Change
NSI: Neurobehavioral Symptom Inventory
QOLIBRI: Quality of Life after Brain Injury
IPAQ: International Physical Activity Questionnaire
PROMIS: Patient-Reported Outcomes Measurement Information System
VSS: Vertigo Symptom Scale
CISS: Convergence Insufficiency Symptom Survey
HIT-6: Headache Impact Test – 6
HADS: Hospital Anxiety & Depression Scale
NDI: Neck Disability Index
mBESS: Modified Balance Error Scoring System
mCTSIB: Modified Clinical Test of Sensory Interaction in Balance
CSMI: Central Sensorimotor Integration
FGA: Functional Gait Assessment
HAM-4-mTBI: 4-Item Hybrid Assessment of Mobility for mTBI
CTC: Complex Turning Course
DVA: Dynamic Visual Acuity
vHIT: Video Head Impulse Test
ANAM: Automated Neuropsychological Assessment Metrics
ROM: Range of Motion
BOSU: Both Sides Utilized
CAB: Community Advisory Board
