# Supplementary material for: Wearable technology to characterize and treat mild traumatic brain injury subtypes: Study protocol for a randomized controlled trial on biofeedback-based precision rehabilitation (SuBTyPE)
Source: PLoS One. 2026 Jan 30;21(1):e0340867. doi: 10.1371/journal.pone.0340867 (PMC12857941; doi:10.1371/journal.pone.0340867)
Supplement: S2 File — (DOCX) [file pone.0340867.s002.docx]

# Minimal Risk Protocol Template

1. Protocol Title

Wearable Technology to Characterize and Treat mTBI Subtypes: Biofeedback-Based Precision Rehabilitation

1. Objectives. Current clinical assessment tools are not sensitive enough to detect and treat some subtle (yet troubling) problems after mild traumatic brain injury (mTBI). In this study we will test a new treatment approach using wearable sensors for feedback during physical therapy that focuses on sensory and motor dysfunction after mTBI. The goal of Aim I is to see if multidimensional real-time biofeedback using novel wearable technology improves outcomes after rehabilitation compared to standard care. The goal of Aim II is to explore the relationship between responsiveness to rehabilitation and subtype classification. The goal of Aim III is to determine if daily life mobility (quality of gait and turning) is impacted differently in people with high V/O deficits

# Background

**Every year 3-4 million people sustain a traumatic brain injury (TBI) in the United States and, of these, up to 90% are considered mild TBI (mTBI) ^1, 2^**. According to data from the Traumatic Brain Injury Center of Excellence, 13,778 service members sustained a mTBI in 2020. The estimated annual cost to society from TBI, including lost productivity, was $60.4 billion ^3, 4^. Balance impairments are a common complaint after mTBI ^5, 6^. Ongoing balance problems are significant contributors to anxiety, difficulty with return to work, and may even underlie the observation that people with a recent history of mTBI are at a threefold greater risk of sustaining a second mTBI and increased risk for musculoskeletal injury ^7-11^.

**Even with rehabilitation, recovery of balance in people with mTBI is challenging and rates of responsiveness to rehabilitation are suboptimal.** In fact, there have been several studies examining the effects of rehabilitation after an mTBI. Two relevant systematic reviews concluded that most of these studies were small and not standardized but that there was weak but promising evidence for vestibular rehabilitation after mTBI ^12, 13^. The authors state that there is a need for larger, high-quality studies to determine efficacy of such rehabilitation for mTBI. Another more recent systematic review that included 12 studies on physical therapy interventions reported that symptom subthreshold exercise may improve symptoms but did not reduce days to recovery and that stand­alone therapies such as vestibular and ocular-motor exercises had limited evidence for improvements ^14^.

**One reason for suboptimal outcomes in rehabilitation may be due to poor performance of the rehabilitation exercises*.*** People with vestibular pathology have impaired perception of head position and movement and may limit their head velocity to minimize symptoms ^15, 16^. We recently published a paper showing that people with mTBI do not move their head as fast or as far when asked to perform walking with head turns, a common task required in daily life ^17^. Vestibular and balance rehabilitation relies on progressively increasing head movement amplitude and velocity during static and dynamic balance tasks while maintaining a stable trunk ^18, 19^. While gait speed is often normal after mTBI, our work showing that head movements during walking with head turns are abnormal, has practical implications for a successful return-to-duty ^17^. A person may compensate for subtle deficits by limiting head movement; this tradeoff should be an important consideration with return to duty, sport, and everyday activities that require both balance and stable gaze. Exercise dosing factors, such as speed and range of head movements, are important and may impact outcomes, but little is known. A clinical practice guideline for vestibular hypofunction reported that there is very little evidence available for exercise dosing recommendations and that researchers should ‘examine the impact of frequency, intensity, time, and type of exercises on rehabilitation outcomes’^20^. Although this is a major gap in our clinical care and the intensity (e.g., velocity of head movement) dose factor is likely a critical aspect of vestibular rehabilitation, exercise intensity is not well defined, nor easily measured. Our group is one of the first to examine the use of wearable sensors to quantify dosing factors during vestibular rehabilitation exercises ^21, 22^.

**Adhering to motor learning principles is essential for successful rehabilitation ^23-25^.** One important principle of motor learning is feedback of performance, yet this important feature is hard to implement objectively in balance rehabilitation. For example, orthopedic physical therapy uses objective measures of joint angles, peak torques and gait speed during task acquisition. To maximize the effects, biofeedback should be immediate and constant at the early stages with progression to slightly delayed and intermittent feedback as the skill improves ^25^. Rehabilitation of balance deficits is more difficult to quantify and to provide feedback while performing a training task. Subtle movements, such as postural sway, head and trunk synchronicity, and head velocity are often not detected visually and therefore not assessed or fed back to patients. Biofeedback has been used for over two decades to improve outcomes of rehabilitation ^26, 27^ but the current approach cannot provide feedback on multidimensional complex movements. For example, biofeedback for balance training typically provides real-­time visual or auditory feedback to practice stability during quiet standing, using a force plate to visualize center of pressure movement ^27^. To date, most balance feedback simply tries to decrease postural sway in static conditions or to improve gait joint motion, without measuring multidimensional movements, like walking with head turns ^28^.

**There are no commercially available biofeedback systems that simultaneously measure multisegmental performance such as head movement amplitude/velocity while concurrently providing objective measures of gait and balance.** Despite evidence that people with mTBI may have both impaired perception of head movement and impaired balance ^6, 17, 29^, we currently have no easy way to quantify impairments nor to provide concurrent feedback during therapy. Computerized dynamic visual acuity testing is available to measure: 1) changes in visual acuity at head velocities associated with the vestibulo-ocular reflex (VOR) and 2) the maximum head velocity the patient can achieve while maintaining accurate vision during a gaze stabilization test ^30^. There are a handful of other clinical systems that can be used to provide feedback on head movement but training is performed in static seated or quiet standing positions in clinical settings only ^31^.

## Our group has developed the first wearable and portable system that combines biofeedback on head and trunk movement while monitoring patients’ dynamic stability during functional tasks (Figure 1) (*W81XWH-17-1-0424*). The system provides the physical therapist and/or patient objective information on the quality of head and trunk movements during training of rehabilitation tasks that can progress from sitting to standing to walking. This system enables physical therapists to evaluate the tradeoff between balance and increasingly complex movements, as well as to teach patients to perform the exercises correctly during increasingly difficult and dynamic conditions. Because of the portability of wearable sensors, the system is intended to be used in any environment where rehabilitation and movement monitoring is needed, including clinical facilities, laboratories, hospitals, outdoors, at-home, or in the field. The system allows the physical therapist to select among a variety of exercises that target balance and vestibular function and has the capability to incorporate motor learning principles such as changing the timing and frequency of feedback, as well as changing rehabilitation goals.


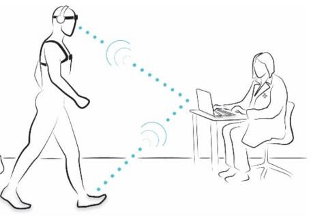


## *Figure 1. Current APDM feedback prototype using a laptop real-time analysis with auditory feedback*

Figure 2

**Another reason for suboptimal rates of responsiveness to rehabilitation after mTBI may be the lack of targeted and precision rehabilitation.** Recent work has identified several mTBI subtypes based on common clinical presentation as *cognitive, ocular-motor, headache/migraine, vestibular, and anxiety/mood* with *cervical strain and sleep* disturbance as associated conditions (Figure 2). Sleep and cervical strain are common with mTBI but do not appear in isolation, therefore are not considered a stand-alone subtype. The concept of mTBI subtypes has been discussed and identified in the literature including an expert panel (Dr. Chesnutt; Co-I on this project), and supported by a systematic review ^32-35^. Correctly identifying the prominent subtypes may be a critical step in determining the most appropriate referral and rehabilitation intervention. However, current subtype classification is primarily based on subjective rating scales ^36^.


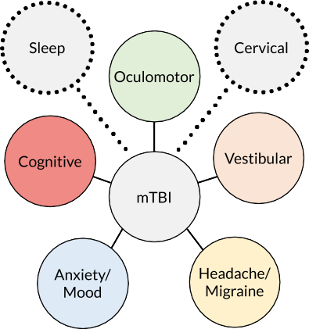


*Figure 2. Five subtypes of mTBI (solid lines). Dashed lines are associated conditions.*

Characterization of published subtypes was based on acute mTBI patients ^34^ but people with ongoing symptoms may have a more complex profile, with overlapping and equally prominent subtypes. **We examined 22 patients from our outpatient OHSU Concussion Clinic using the Concussion Symptom Subtype Inventory (CSSI) ^37^ profile screen. We found that only 12% of the patients seen in our clinic over the past month with moderate symptoms had one clear primary subtype. Twenty-four percent had 2 subtypes of equal severity and 65% had greater than 3 subtypes. These people were, on average, 11.2± 20.1 months from injury with an average age of 38 ±16 years. It is currently unclear how to use the available scales to characterize subtypes on more complex patients who may have high symptom burden across subtype domains. This project will explore how best to characterize subtypes using multiple outcome measures to help direct patient care. The most appropriate mTBI subtypes to benefit from physical therapy are sensorimotor subtypes including vestibular and/or ocular-motor but these measures are under-represented in subjective rating scales ^38^.** A recent study that examined the 8 most commonly used questionnaires found that vestibular and ocular-motor (V/O) subtypes were minimally represented in the questions presented on the questionnaires. Specifically, ocular-motor was 11% and vestibular was 19% of the questions in the scales ^38^. Therefore, using a subjective approach to determine primary subtypes may lead to missed opportunity for rehabilitation. Alternatively, many people may be inappropriately referred to physical therapy (i.e., cognitive subtype) who would benefit more from a referral to neuropsychology or speech and language therapy. Anecdotally and based on our pilot data (see below), referrals to physical therapy that may be inappropriate can lead to low responder rates. This proposal will attempt to better define the subtypes of *vestibular* and *ocular-motor* (V/O) to improve identification leading to improved rehabilitation outcomes.

**Measures of sensorimotor impairment are often subjective and easily overlooked as a treatable deficit.** For example, the most frequently administered clinical balance test after mTBI is the Balance Error Scoring System (BESS) in which people are asked to stand in varying conditions (i.e., eyes closed on a firm or foam surface) to challenge postural stability ^39^. Errors, such as loss of balance, are counted subjectively by the clinician. However, studies have reported that the BESS has a high degree of subjectivity and provides only limited, low-resolution information about the balance control system and does not provide a sensitive scale on which to judge progress or deficits ^40, 41^**.** Sub-optimal psychometric properties (sensitivity 34%–64% to detect mTBI) have also been reported for the BESS and are likely due to the subjectivity of the measurement ^40, 41^. Clinical balance tests with limited clinimetric properties may lead to lower detection rates and make it difficult to identify those who might benefit from physical therapy. More objective measures of balance may help identify people with impaired balance due to sensorimotor deficits (i.e., vestibular and/or ocular-motor) in which a targeted physical therapy intervention would help recovery.

**Recent evidence suggests that objective measures of function are a critical part of assessment after mTBI.** In the subtype classification, gait and balance deficits fall under the *vestibular* subtype. Such deficits are often subtle, yet very troubling, and can often only be detected objectively using instrumented measures such as force plates and wearable sensors to measure postural sway, central sensorimotor integration, and gait parameters ^39-42^. Our laboratory has been on the forefront of developing objective measures to improve detection of deficits to facilitate targeted and precision rehabilitation. Our work has provided evidence that instrumenting common balance tests with body-worn inertial sensors can better differentiate people after mTBI compared to subjective measures of balance ^42-44^. While objective measures have been deemed essential for assessing deficits after mTBI, routine clinical care typically does not include such objective measures. Wearable sensors have the potential to change health care outcomes due to their objectivity, increasing utility, accessibility and affordability.


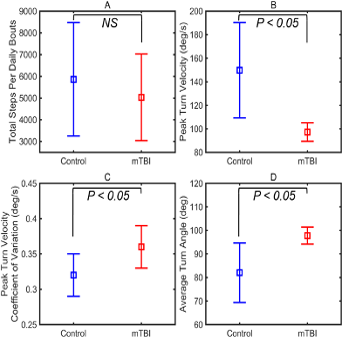


*Figure 4. Control and mTBI subjects had similar daily step counts over 7 days (A) but mTBI subjects had slower peak turning velocities (B), more variable peak turning velocities (C), and larger average turning angles (D).*

**Body-worn technology, which can now automatically quantify hundreds of gait and balance measures in supervised and un-supervised settings, may facilitate mTBI subtype classification**. Most body-worn sensors measure *quantity* of activity, such as number of steps or activity levels. However, in partnership with *APDM Wearable Technologies*, our group has developed a novel, body-worn inertial system to characterize *quality*, such as abnormal turning patterns ^45-47^. Our novel instrumented socks (Figure 3) can be worn during daily life for continuous monitoring. We recently demonstrated that quality, but not quantity, of daily life activity at home is impaired in people with mTBI compared to healthy control participants ^48^, and particularly turning mobility (Figure 4). *Therefore, we are uniquely positioned to assess daily functional mobility impairments in people with mTBI with the goal of improving subtype classification.* Functioning optimally at home and in everyday life is the goal of recovery after mTBI. Our goal is to optimize assessment across subtype classification and generate evidence for novel outcome measures that predict real-world function and facilitate recovery.


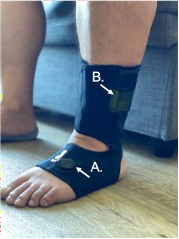


*Figure 3.* *Instrumented socks with inertial sensors on top of foot (A) and battery on the side (B).*

**There has been little to no work in determining the strength of the relationship between clinical subtypes and objective measures, an area of known importance in mTBI.** In this proposal, we will use objective measures to address potential obstacles for successful rehabilitation after mTBI. We will use multidimensional, real-time biofeedback to improve quality of performance during rehabilitation and use state-of-the-art wearable technology to identify V/O subtypes that may be more responsive to rehabilitation.

**Rigor of previously related funded work.** This proposal is a natural continuation of our previous funding that used objective measures to improve assessment and precision rehabilitation after mTBI. In 2012 we were awarded a pilot grant (NIH; *Center for Translation of Rehabilitation Engineering Advances and Technology; King PI*) to develop a protocol for instrumented balance testing for people with chronic post-concussion syndrome ^42^. From there, we moved the instrumented balance testing to on-site assessments at 6 local universities to explore the usefulness of wearable sensors in acutely-injured athletes and to measure the trajectory of balance recovery after mTBI (*NIH; Oregon Clinical Translational Research Center (OCTRI) KL2 Award and National Institute of Health KL2TR000152; R21 NIH R21HD080398; King PI*). We received several Department of Defense (DoD) grants to 1) investigate central sensorimotor integration and vestibular deficits in people with mTBI (*King PI-W81XWH-15-1-0620*), 2) investigate optimal timing of rehabilitation and to develop a system using wearable sensors to facilitate vestibular rehabilitation after mTBI (*King PI- W81XWH-17-1-0424*), and 3) to develop and test ecologically valid assessments of dual-task testing (with 2 other academic and 3 military sites) (*King PI-W81XWH-18-2-0049*).

We have had several SBIR/STTR grants in collaboration with *APDM Wearable Technologies* producing wearable sensors for documenting physical movements including: a Phase 1 DOD contract (*W81XWH19C0125*) to develop a ruggedized wearable sensor system to assess movement dysfunction in a field environment and to guide clinical and return-to-duty decision-making, a Phase II SBIR *(R44AG055388; NIA*) to assess continuous monitoring of mobility in daily lives of people with neurological disease, a Phase I-II SBIR (*AG044863; NIA*) to continuously measure functional mobility in older people with fall risk, a Phase I-II SBIR (*AG056012; NIA*) to develop and commercialize a gait biofeedback system for physical therapists to provide biofeedback for gait training in people with gait impairments, and a Phase II SBIR (*HHSN261201600067C; NCI*) to develop a portable clinical system to quickly and automatically obtain objective measures of balance and gait impairments. APDM and OHSU completed a Phase I-II STTR (*R42 HD071760; NCMRR*) that enabled APDM to develop and commercialize its Mobility Lab system with protocols and reports for the Short-Instrumented Test of Mobility to provide a validated, objective fall risk evaluation. Simultaneously, our team has been involved in exploring the clinical profiles of people after mTBI and Dr. Chesnutt (Co-I) has been selected as a member of the concussion subtype national expert panel that delineated concussion subtypes supported by a systematic literature review ^34, 35^. The proposed project uses the technical advances and scientific understandings made in these projects for this Level 2 Clinical Trial Award.

# PRELIMINARY STUDIES

**Aim I. Multidimensional, real-time biofeedback for rehabilitation.** The goal of Aim I is to determine if multidimensional real-time biofeedback using novel wearable technology improves outcomes after rehabilitation compared to standard care. With previous funding from the Department of Defense (*W81XWH-17-1-0424*), we have developed a novel, wearable sensor-based system that can deliver multidimensional real-time biofeedback during complex activities and exercises that require precise head/trunk movement while maintaining balance during standing and walking with a user-friendly interface (Figure 5). It is common to decrease head motion to minimize exacerbation of symptoms but this compensatory strategy will not be optimal when a person returns to duty or to sport. The feedback delivered by the system is instantaneous, adaptable by the physical therapist, and provides multiple objective measures concurrently during ecologically-relevant, functional tasks.


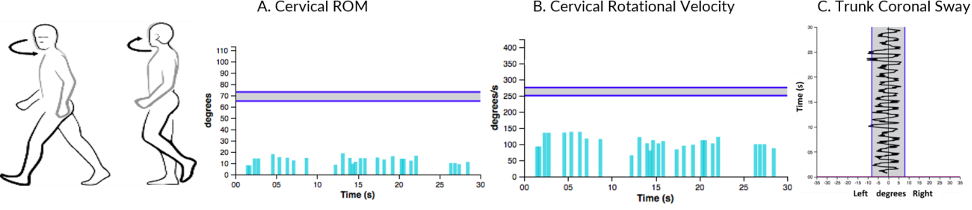


Figure 5. Screenshot of a participant performing walking with head turns; demonstrating that A) cervical range of motion (ROM) and B) velocity are reduced compared to the normal, target zone (horizontal grey line). Panel C shows that despite slowed head movements, the person’s gait is stable and within the mediolateral target zones determined by healthy control data.

**This novel system can detect the tradeoff between speed/amplitude of head movement and stability, a critical consideration in the readiness for return to duty.** The screenshot in Figure 6 shows that a person may have very slow and small head movements yet be quite stable in quiet stance (red in stabilogram; panel C). However, when the patient is provided feedback (see dashed line at 12 seconds) to increase head speed or amplitude, they show quantifiable signs of imbalance (black stabilogram; panel C). The gray horizontal shaded areas in A and B show healthy normative ranges and the end-goal for people with mTBI. Most available biofeedback systems measure either head motion or postural sway, but not both simultaneously; this system concurrently measures multiple domains which means individuals can train using functional tasks more reflective of the concurrent demands of daily life.


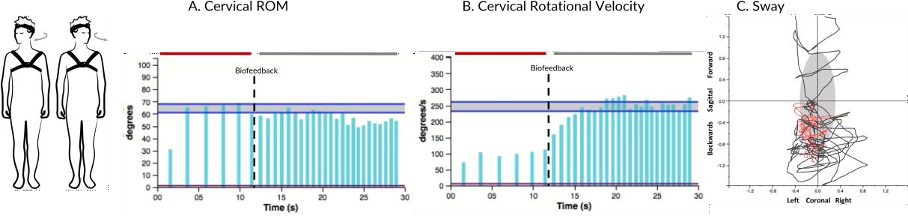


*Figure 6. Gaze stabilization task requiring head movement at a specified speed and amplitude. This screenshot shows performance tradeoff of a person standing while performing head turns. (A) Cervical range of motion (ROM) was normal but speed (B) was at suboptimal levels prior to biofeedback, although postural sway (C) was stable (red line). Once feedback to improve head speed and maintain amplitude was initiated (after dashed line), postural sway increased (C-black line) to outside of the target goal zone of healthy normative values (grey ellipse panel C).*

## This feedback system provides immediate feedback on correct task performance. Figure 7 shows the capability of this system to detect poor performance on a rehabilitation task. In this example, the person is instructed to maintain a stable gaze, while turning the head (gaze stabilization exercise). In standing gaze stabilization, there should be a dissociation between the head and the trunk. But, in order to minimize symptoms associated with increased stimulation to the vestibular system (i.e., head movement), a person often moves the trunk together with the head as a compensatory strategy. The screenshot in Figure 7 shows that this person initially had excessive trunk motion but with feedback (dashed lines at 15 s) could alter this movement pattern to isolate head movement.


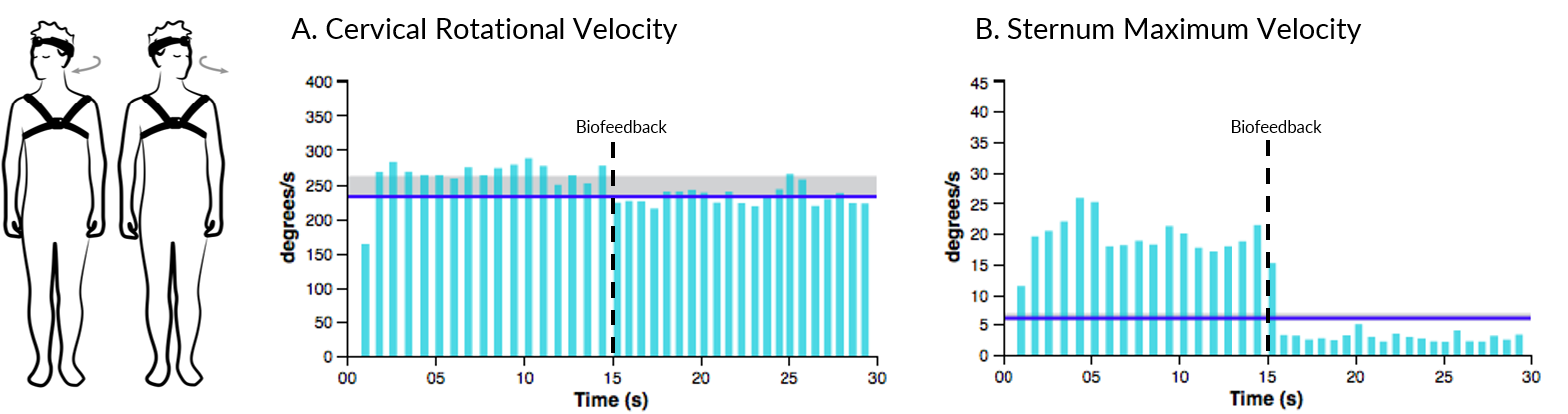


*Figure 7. Screenshot demonstrating that initially this person had excessive trunk motion, but after feedback was able to move the head* *while keeping trunk still during a gaze stabilization exercise.*

## This multidimensional real-time biofeedback is feasible in the clinic. We have preliminary data showing that this system is feasible and that physical therapists think it would be useful in the clinic. Using the System Usability Scale ^49^, which asks about perceived usability and usefulness, we found that 3 different physical therapists who treat people with mTBI had favorable opinions of the system. After using the system with several different patients with mTBI during their clinical visit, the average System Usability Scale score was 80 ± 3.5, a calculated score that falls between ‘good’ and ‘best imaginable’ ^49, 50^. See Table 1 for specific item questions.

| **Questions rated from 1 (strongly disagree) to 5 strongly (agree)** | **Mean ±** **SD** |
| --- | --- |
| I think that I would like to use this system frequently | 4.7 ± 0.6 |
| I would imagine that most people would learn to use this system very quickly | 4.3 ± 1.2 |
| I found the various functions in this system were well integrated | 4.3 ± 0.6 |
| I thought this system was easy to use | 4.0 ± 0.0 |
| I felt very confident using this system | 3.3 ± 0.6 |
| I think that I would need assistance to be able to use this system | 2.3 ± 1.5 |
| I needed to learn a lot of things before I could get going with this system. | 2.3 ± 0.6 |
| I found this system unnecessarily complex | 1.7 ± 0.6 |
| I thought there was too much inconsistency in this system. | 1.7 ± 0.6 |
| I found this system very cumbersome/awkward to use. | 1.0 ± 0.0 |

*Table 1 Results from the System Usability Scale for trial of our novel multidimensional real-time biofeedback system in our outpatient concussion clinic.*

**Aim II. Responsiveness to rehabilitation; objective measures to identify vestibular and/or ocular-motor subtypes.** The goal of Aim II is to explore the relationship between responsiveness to rehabilitation and subtype classification. We have pilot data showing that mTBI subtyping is not routinely used in clinical practice for referrals and this could result in inappropriate referrals to physical therapy, as well as unproductive use of time and money on a treatment that may not help. Further, the time spent in the wrong type of rehabilitation is wasting a critical window of recovery where the correct, targeted treatment could help. We will explore how best to classify V/O subtypes using objective measures with the goal of improving responsiveness to rehabilitation.

**Current practice does not consistently use subtype classification for referrals - better classification with objective measures may change this pattern.** We retrospectively examined 22 patients from the OHSU Concussion Clinic to determine if mTBI subtype related to referral patterns for physical rehabilitation. Ideally, we would expect to see the patients with high V/O deficits consistently being referred to physical therapy, rather than the other subtypes (i.e., anxiety/mood and cognitive). We found that 15 people reported symptoms of vestibular or ocular-motor complaints on the CSSI V/O categories; of these, only 8 were referred to physical therapy. Of the 7 who had no V/O symptoms, 3 were referred for physical therapy. Of these 3, none were classified with cervical signs/symptoms that could explain the referral. This potentially inappropriate pattern of referral could result in poor outcomes and low responder rates after rehabilitation.

**Responsiveness to rehabilitation increases when only people with objective measures of V/O deficits are included.** In people with subacute mTBI who went through a 6-week, multimodal physical therapy program, we calculated responsiveness to intervention using the top 2 levels of the Patient Global Impression of Change (PGIC) (defined as a ‘definite’ improvement and ‘made a world of difference’ on a 7-point scale) ^51, 52^. We found that when considering the group as a whole (n=72), regardless of subtype, 74% were considered ‘responders’ to rehabilitation; leaving a sizable number of people (26%) who did not improve. This poor responder rate is not uncommon for physical therapy interventions ^14^. However, when we considered only those with objective V/O laboratory measures (n=14), the responder rate went up to 93% (Figure 8). Of note, this classification was based solely on laboratory (not self-report) measures of V/O function.


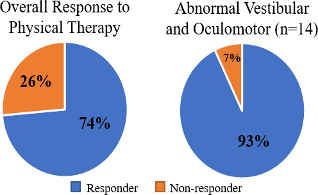


*Figure 8. Responder rate after 6-weeks of rehabilitation after mTBI (King, pilot data). Left graph includes all subtypes and right graph only the subtypes with abnormal vestibular and/or oculomotor function.*

**Patient-reported measures of V/O symptoms may not relate to Clinical or Instrumented Assessment so both subjective and objective measures may be critical in classifying mTBI subtypes.** We analyzed preliminary data on 12 people who completed the CSSI ^37^ as well as other V/O measures that fall under Patient reported, Clinical and Instrumented. Figure 9 shows the highest correlations were between CSSI V/O subscores and other Patient-reported measures. There were very few to no significant correlations between CSSI V/O subscores and Clinical or Instrumented V/O Assessments. The lack of relationship between Patient-reported V/O outcomes and Clinical and Instrumented V/O measures highlight the importance of including both subjective and objective measures in our assessment after mTBI.


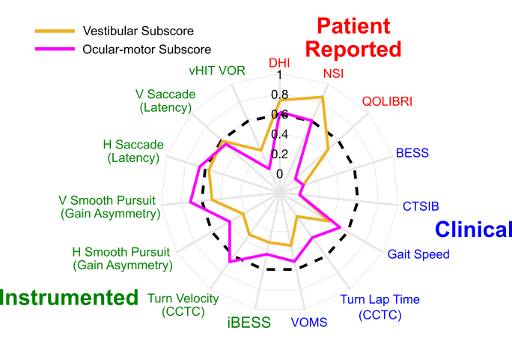


*Figure 9. Radar plot showing poor correlations between CSSI V/O subscores and Clinical and Instrumented Assessments in the vestibular (Orange) and ocular-motor (Pink). The dashed line represents significance at 0.05.*

## Aim III. Daily life mobility in people with V/O subtype. The goal of Aim III is to determine if daily life mobility (quality of gait and turning) is impacted differently in people with high V/O deficits. We have compelling preliminary data suggesting that people with high V/O deficits have more impaired quality of daily life mobility compared to people with low V/O deficits. We have already published that people with mTBI (without considering subtype) have different quality of movement compared to healthy control participants during daily life ^48, 53^. We will explore if daily life mobility is different in people with high V/O deficits compared to low V/O deficits.

## People with impaired vestibular and ocular-motor function had impaired quality of turning during daily life compared to people without deficits in these areas. Figure 10A shows that the measures of *quantity*, such as steps per day and activity rate, are similar in people with and without V/O deficits, whereas Figure 10B shows clear differences in daily turning in people with versus without V/O deficits. Vestibular and ocular-motor function was measured using objective laboratory testing. People with documented V/O deficits demonstrate smaller turn angles, less variability in turns and lower peak velocity of turns in 7 days of daily life.


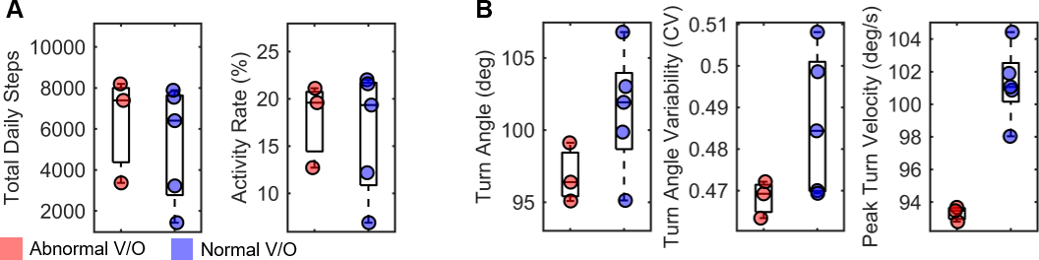


*Figure 10. Continuous home monitoring over 7 days in 8 people with mTBI showing A) people with mTBI who had abnormal vestibular and/or ocular-motor function had similar activity rates (quantity) including total daily steps and daily activity rate as those without abnormalities in those domains while B) people with vestibular and/or ocular-motor abnormalities had different quality of movement including daily turn angles, turn angle variability and peak turn velocity compared to those without abnormalities in those domains.*

# OBJECTIVES/SPECIFIC AIMS

# *Objectives:* Our long-term goal is to improve the assessment and rehabilitative treatment of patients with mTBI with a focus on integrating objective measures for identifying and treating V/O subtypes. Our central premise is that objective measures are a critical, yet missing, component of a comprehensive assessment and treatment plan after mTBI. We believe that rehabilitation, commonly prescribed after mTBI, will benefit from integrating immediate and objective information on complex sensorimotor task performance using a novel, wearable, sensor-based system recently developed by this team. Further, we believe that integrating objective measures of function, including daily life mobility, will provide an important aspect of subtype identification after mTBI and will ultimately facilitate precision rehabilitation. While it is well accepted that objective measures are essential in identifying deficits after mTBI, subtype identification is currently based solely on symptom reporting ^36, 37^. Within this clinical trial, we will explore whether using novel, wearable technology can improve rehabilitation by integrating multidimensional, real-time biofeedback during rehabilitation and by using objective measures to better identify people with sensorimotor deficits (as associated with V/O subtypes). These sensorimotor subtypes are more likely to have higher response rates to physical rehabilitation, compared to those with non-sensorimotor deficits, such as anxiety/mood and cognitive subtypes. Further, we will explore whether quality of daily mobility (i.e., continuous monitoring of gait and turning), differs in people with high V/O deficits. We will focus on whether objective measures of V/O function provide insight into responsiveness to rehabilitation and explore the relationship between subjective and objective measures of V/O function to improve subtype classification.

**Aim I. Multidimensional, real-time biofeedback for rehabilitation:** To determine if multidimensional real­time biofeedback using novel wearable technology that measures head/trunk and balance/gait improves outcomes after rehabilitation compared to standard care. *We hypothesize that augmenting rehabilitation with wearable sensors to simultaneously measure and provide feedback on head, trunk and balance/gait will improve outcomes.* For Aim I, 100 people with mTBI from 2 sites (Oregon Health & Science University (OHSU) and University of Utah (UU)) will be randomized into either standard care or standard care augmented feedback using wearable sensors. People will be tested before and after a 6-week (2x/week) rehabilitation program. Our primary outcome will be the Patient Global Impression of Change (PGIC) Scale ^52^. Secondary outcomes will be structured according to Subjective Patient-reported (including qualitative measures), Clinical Assessment, and Instrumented Assessment measures (Table 4). We will measure covariates to help interpret rehabilitation efficacy. We will: 1) determine if multidimensional real-time biofeedback improves efficacy of rehabilitation after mTBI and 2) summarize perceptions from the treating physical therapists to help inform next steps for clinical implementation of head/trunk/balance biofeedback.

**Aim II. Responsiveness to rehabilitation; objective measures to identify V/O subtype**: Aim II is a separate analysis based on the data collected in Aim I. Here, we will explore: a) the responsiveness to rehabilitation by level of V/O deficit and b) the strength of the relationship between Patient-reported and Clinical and Instrumented Assessments of V/O measures. To focus on people with prominent V/O deficits, we will group people according to severity of V/O symptoms; moderate to severe (V/O HI) and minimal (V/O LO). Groups (V/O HI and LO) will be based on the Concussion Clinical Profile Screen (CP Screen) since this subtyping tool has been validated and includes both vestibular and ocular-motor domains ^36^. In this way of categorizing our groups, we will have more severely impacted people from V/O deficits to compare with less V/O deficits that would include people from *cognitive, headache/migraine, and anxiety/mood cognitive* subtypes. *We hypothesize that a) people with V/O HI will demonstrate a higher rate of responsiveness compared to those people with V/O LO and b) there will be only a small to moderate relationship between Patient-reported and Clinical and Instrumented Assessments that relate to V/O measures.* For Aim II, we will use the data collected at baseline from Aim I, prior to rehabilitation, and a “*responder”* to rehabilitation will be defined using the highest 2 categories of the PGIC ^51^ (Figure 15). To explore the relationship between Patient-reported and Clinical and Instrumented Assessments, we will examine the strength of correlations across all V/O measures.

**Aim III. Daily life mobility in people with V/O subtype:** To: a) determine if daily life mobility (quality of gait and turning) is impacted differently in people with high V/O deficits and b) calculate healthy normative data for daily life mobility measures in active duty military service members. *We hypothesize that gait and turning quality, measured during 7 days of daily life, will be worse in people with high V/O deficits.* We hypothesize that people with V/O HI will have worse quality of mobility during daily life (turn angles, variability and velocity) than those with V/O LO. For Aim III, 50 people from Aim I, divided equally with V/O HI and V/O LO, will wear instrumented socks (*APDM Wearable Technologies*) (Figure 3) over a 7-day period. Since the instrumented socks are currently a prototype provided by *APDM Wearable Technologies*, our supply will allow only for this subset of 50 participants. Daily life mobility (quantity and quality) will also be collected on 40 healthy active duty service members over 7 days at Fort Sam Houston (FSH) to facilitate next steps in using continuous monitoring as an outcome measure after mTBI in the military.

Table 2 Overview of Study Aims and Milestones

| **Aim** | **Focus** | **Protocol** | | **Participants** | | **Milestones** | |
| --- | --- | --- | --- | --- | --- | --- | --- |
| **I** | Multidimensional real-time biofeedback using wearable sensors during rehabilitation | | Aim 1: Randomized to biofeedback or standard care | | 100 people with mTBI:  OHSU (n=50)  UU (n=50) | | 1. Determine if multidimensional real-time biofeedback improves efficacy of rehabilitation 2. Summarize qualitative assessments from the treating physical therapists to help inform next steps for clinical implementation |
| **II** | Responsiveness to rehabilitation and V/O subtype classification | | Aim II: Calculate percentage of responders to rehabilitation across V/O subtypes; explore correlations between types of measures used for V/O subtype classification | | All 100 mTBI subjects from Aim I | | 1. Calculate responsiveness to rehabilitation across severity of V/O deficits, using objective measures of V/O deficits 2. Explore the strength of correlations between subjective and objective measures of V/O function |
| **III** | Continuous monitoring of daily life function with wearable sensors | | Aim III: A subset from Aim I will instrumented socks for 7 days  Healthy military normative data | | 50 people with mTBI:  OHSU (n=25)  UU (n=25)  40 healthy control active duty military FSH (n=40) | | 1. Characterize V/O subtypes based on daily life mobility 2. Calculate healthy normative data for active duty military service members |

**STUDY DESIGN/STATISICAL PLAN/DATA ANALYSIS**

*Participants.* Participants will include 100 civilians with persistent symptoms from mTBI and 40 active duty, service members as healthy controls. Two civilian/academic sites (OHSU and UU) will test civilians while one military site (FSH) will test military service members for daily life home monitoring (Aim III FSH).

*Recruitment.* Our primary sources for recruitment of civilians with mTBI will be the OHSU Concussion Clinic (see Letter of Support; Dr. Chesnutt), and the University of Utah Outpatient Rehabilitation Services Orthopedic Center and the Balance and Mobility Clinic (See Letters of Support; Drs. Ballard, Ward, and Cohee). Active-duty military control participants will be recruited at Fort Sam Houston from personnel assigned or attached to the U.S. Army Medical Center of Excellence (MEDCoE) and the Military Education Training Campus (See Letter of Support; Dr. Douglas). Recruitment will occur by word of mouth, flyers and posters distributed around these facilities and briefings to assigned personnel.

*Inclusion criteria*. For all Aims, participants may be either civilians, active duty military, or Veterans, and must: 1) have a diagnosis of mTBI based upon VA/DoD criteria ^54, 55^, 2) be between 18-50 years old, 3) be outside of the acute stage (> 3 weeks post-concussion) but within 3 years of their most recent mTBI and still reporting symptoms, and have at least some measurable deficit in V/O categories based on CP Screen (See justification below).

Aim III healthy controls: Participants must be active duty military service members with no recent (< 3 years) history of mTBI and no residual symptoms from any remote mTBIs. *Exclusion criteria for all Aims*: Participants must not: 1) have had or currently have any other injury, medical, or neurological illness that could potentially explain balance deficits (e.g., CNS disease, stroke, epilepsy, greater than mild TBI, lower extremity amputation, recent lower extremity or spine orthopedic injury requiring a profile) 2) meet criteria for moderate to severe substance-use disorder within the past month, as defined by DSM-V, 3) display behavior that would significantly interfere with validity of data collection or safety during study, 4) be in significant pain during the evaluation (> 7/10 by patient subjective report), 5) be a pregnant female (balance considerations), 6) have past history of peripheral vestibular pathology or ocular motor deficits, or 7) be unable to abstain in the use medications that might impair their balance for 24 hours in advance of testing.

*Justification for CP Screen V/O cut-offs:* The concept of individuals with mTBI having one primary subtype was developed on people seen in the acute stage of mTBI. People who do not recover quickly and have a longer time frame since diagnosis often develop a more heterogeneous profile. These are typically the people we see in our rehabilitation clinic and therefore, it was not feasible to use a primary subtype as a grouping variable. This project proposes an alternative method of categorizing individuals with vestibular and/or ocular clinical subtypes based on their CP Screen. People will be categorized as no V/O deficits (excluded from study), low V/O deficits (V/O LO) and high V/O (V/O HI) deficits. To be included in the study, people will be required to have some level of V/O deficits to justify rehabilitation. Our expectation that individuals with high V/O scores would be expected to have stronger response to physical rehabilitation. The chosen cut-offs were based on a pilot study we conducted of 12 healthy individuals with no recent (< 3 years) history of mTBI and no residual symptoms from any remote mTBIs (the same inclusion criteria for Aim III healthy controls).

Groups – Based on Vestibular or Ocular scores or Both

1. Exclude 0.0 to 0.6 (3 or less out of max of 15)
2. V/O LO 0.61 to 1.99 (4 to 9 out of max of 15) corresponds to in general mild
3. V/O HI 2.00 to 3.00 (10 to 15 out of max of 15) corresponds to in general moderate to severe symptoms

To help determine if our cut-offs relate to the patient’s perception, we will ask all participants which clinical subtype primarily accounts for their symptoms and if they perceive the symptoms in their self-identified predominant profile as low or high. We acknowledge that the CP Screen scores may not be linear, and that even healthy individuals did not have a score of zero. The qualitative reports of the patients will be considered with the CP Screen, and cut-offs will be adjusted to ensure that the intent of our inclusion criteria is met.

**Aim I: Multidimensional, real-time biofeedback for rehabilitation.** To determine if multidimensional biofeedback using novel, wearable technology that combines head and trunk movement with dynamic balance improves outcomes after rehabilitation compared to standard care.

*Rationale.* Rehabilitation therapies have produced disappointing results in many patients with prolonged symptoms after mTBI ^56^. Our underlying premise is that objective measures are a critical, yet missing, component of a comprehensive assessment and treatment plan after mTBI. We believe that rehabilitation, commonly prescribed after mTBI, will benefit from integrating immediate and objective information on complex task performance using a novel wearable sensor-based protocol recently developed by our team. Current limitations in mTBI rehabilitation include administering specific rehabilitation exercises without knowing appropriate dosing metrics (i.e. velocity and amplitude of head movement) and lack of objective measures of performance. This approach does not follow motor learning principles and may not maximize neuroplasticity. Aim I will determine if using objective measures of head/trunk/balance exercise performance with immediate feedback on complex movements will improve rehabilitation after mTBI.

*Study Design.* This is a single-blinded, randomized controlled trial to determine if multidimensional real-time biofeedback improves outcomes after rehabilitation. Participants with mTBI will be randomized into 6 weeks of multimodal rehabilitation consisting of either 1) standard care or 2) standard care plus biofeedback (Figure 11).*Blinding and Randomization.* After baseline testing, each participant will learn their group assignment by opening a sealed, sequentially-numbered envelope that contains the randomly-determined group assignment. Because sensorimotor deficit (V/O) is central to several of our hypotheses, we will use a stratified block randomization scheme. This approach will be stratified using V/O HI and V/O LO so that both rehabilitation interventions will get approximately equal number of participants with the same severity of V/O deficits. Block randomization will help ensure equal numbers in the two arms, and we will vary block sizes (2, 4, 6 or 8) to help avoid selection bias. The participants will not be told which intervention is favored, the groups will be trained at separate times, and the researchers administering the pre and post tests and analyzing the data will be blinded to group assignment.


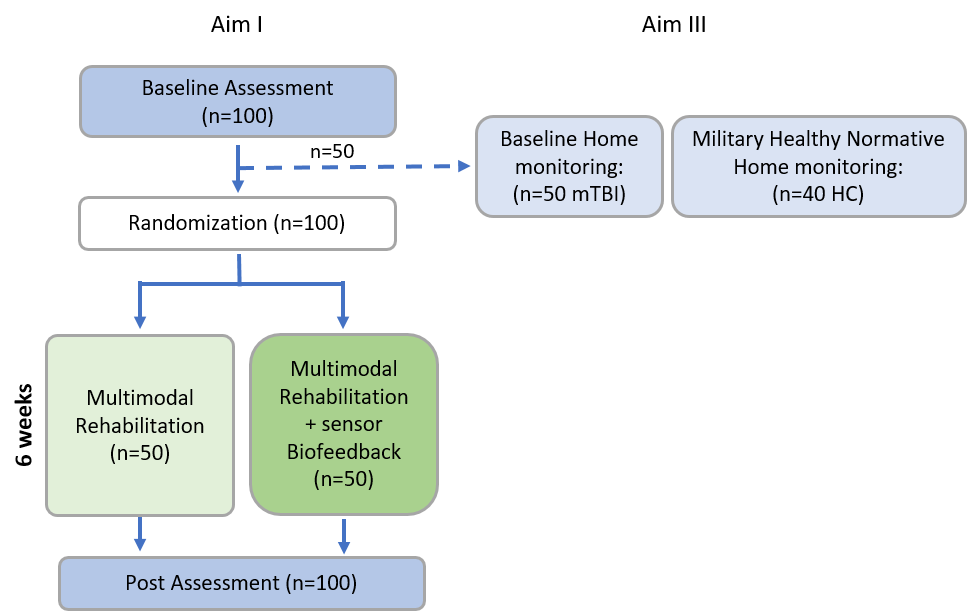


Figure 11 Study design

*Power and sample size considerations.* To power this Aim, we used pilot data on participants with mTBI who received physical rehabilitation for balance deficits. This data, obtained during the development phase of the biofeedback system (*W81XWH-17-1-0424*), was based on using wearable sensors to measure multidimensional movement but the feedback was not real-time. Rather, it was delivered at the next physical therapy session. This step was critical to development of the current real-time system. In a group of 43 participants with mTBI, half of the participants received standard care physical therapy and the other half used wearable sensors during exercise at home and were provided feedback at the subsequent session. At the conclusion of rehabilitation, participants rated their perception of change using the PGIC after intervention. We found that the participants who provided sensor data to the physical therapist on objective measures of performance had a mean PGIC score 0.5 points (SD=1.3) higher than the standard care group. In the proposed study, we anticipate we will observe a larger mean difference (0.75) because the sensor group will receive immediate feedback. Also, we anticipate a smaller standard deviation because of more consistent therapeutic results (SD=1). Using a Mann-Whitney U test, we estimate that we will have 90% power to detect a mean difference of 0.75 (SD=1) using a two-sided test, alpha at 0.05, and with 40 participants per group. This sample size takes into account an anticipated dropout rate of 25%. Calculations were computed using PASS 2019 ^57^.

*Study Intervention.* We designed a multimodal rehabilitation approach based on the current recommendation from the concussion clinical practice guideline (CPG) that includes a standardized approach to target the common dysfunctional areas after mTBI; cervical, autonomic, vestibular/ocular-motor and motor function ^58-61^. We will individualize the protocol based on the impairments each participant demonstrates during the physical therapy assessment. Each domain will have several areas of focus that will be targeted and advanced based on participant tolerance. The physical therapist can determine where to focus each treatment based on participant performance and symptoms. If there are no impairments or functional limitations present, the physical therapist can minimize time spent in that domain. We will document the components of each treatment session for all participants so that we can summarize the components and progressions of each of the intervention groups. Both groups will use the same rehabilitation interventions (Table 3) with the exception of real-time biofeedback used during rehabilitation for cervical, vestibular, ocular-motor, and motor function impairments. Each participant will come to physical therapy 2 times per week for 1-hour sessions for 6 weeks. The rehabilitation will consist of 4 exercise ‘domains’ (Table 3) with several areas of progression built in per task. Each exercise will be challenged with increasing task demands (static to dynamic), speed/amplitude increases, sensory changes (i.e., eyes closed or soft surface) and/or cognitive (dual task training) additions.

Table 3. Summary of exercise tasks, balance domains, safety modifications, progressions and exercise goals

| **Domain** | **Areas of focus** | **Multidimensional Biofeedback** | **Challenges** | | | | **Goals** |
| --- | --- | --- | --- | --- | --- | --- | --- |
|  |  |  | Velocity | ROM | Sensory | Dual task |  |
| Cervical | 1. Manual therapy  2. Stretching exercises  3. Strengthening exercise  4. Sensorimotor control | - Cervical ROM |  | x | x |  | Restore ROM, reduce pain, improve proprioceptive input and strength.  Potentially address headaches/dizziness |
| Autonomic | 1. Cardiovascular exercise based on Buffalo treadmill test |  |  |  |  | x | Improve overall symptoms, return to sport/activity, aide in neurological recovery |
| Vestibular/  ocular-motor | 1. Dix Hallpike  2. Oculomotor exercises  3. Gaze stabilization  4. Visual motion sensitivity | - Cervical ROM/velocity - Trunk ROM/velocity - Balance/gait stability | x | x | x | x | Reduce dizziness, improve balance |
| Motor function impairments | 1. Static balance  2. Dynamic balance  3. Motor coordination and control  4. Dual tasks | - Cervical ROM/ velocity - Trunk ROM/velocity - Balance/gait stability | x | x | x | x | Improve motor function and functional status |

*Mobility Lab for multidimensional real-time biofeedback*: The system we will use for the intervention was developed with previous Department of Defense funding (*W81XWH-17-1-0424*) by several of the team members in this current proposal. The system uses synchronized inertial sensors (Opals by APDM; Dr. El-Gohary Principal engineer at APDM and Co-I on project). The Opals have 3D accelerometers, gyroscopes, and magnetometers. The Opal sensors will be worn on the head, lumbar, and feet using elastic Velcro bands during the rehabilitation sessions for common cervical, vestibular/ocular-motor and motor function exercises. The inertial data on head ROM/velocity, trunk ROM/velocity and stability will be collected, synchronized, and wirelessly transferred to a nearby laptop computer for analysis and feedback visualization. The physical therapist can use this instrumented data to provide either immediate or delayed feedback to improve both the quality of movement and stability based on motor learning principles. The physical therapist will be able to select among a variety of exercises, advance level of difficulty, progress target zones and change the timing and type of feedback (visual vs auditory) based on patient performance and symptoms (Figure 12).


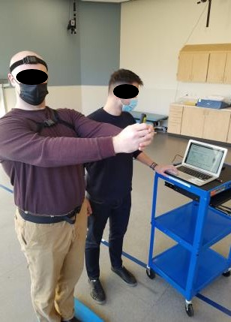


Figure 12 Participant and physical therapist with multidimensional real-time biofeedback

## *Outcomes.* The primary outcome measure for Aim I is the PGIC. Secondary outcome measures will satisfy the Common Data Elements (CDE) as provided by the National Institute of Neurological Disorders and Stroke (NINDS) CDE TBI Project ^62^ and will be categorized according to patient Patient-reported, Clinical Assessment, and Instrumented Assessment. In accordance with CDE recommendations, several measures fall under the core, highly recommended or recommended measures ^63-65^. The forms selected for use in this study are also common data elements in Federal Interagency Traumatic Brain Injury Research (FITBIR) and will allow for easy data management. For measures that are instrumented with wearable sensors, Opal sensors (APDM) will be used to calculate objective measures of mobility (balance, gait and turning) and will provide both commercially available metrics from the software (Mobility Lab) as well as novel metrics based on our own algorithms ^66, 67^. Wireless synchronization, unique to the APDM Opal sensors, allows precise temporal binding of data across sensors. The outcome measures are summarized in Table 4.

***Patient-reported Assessment:*** The *Patient Global Impression of Change (PGIC)* is a one-question measure on a seven-point Likert scale, in which the external judgment of meaningful change is made by the patient ^52^. In 2014, the PGIC was chosen, by DoD stakeholders of the Office of Outcomes and Assessment at the Defense and Veterans Brain Injury Center, as one of the two core measure for mTBI health care outcomes, in regards to benefit from treatment ^68^. This is the primary outcome measure. The *Dizziness Handicap Inventory (DHI*) is a 25-item self-assessment inventory that evaluates the self-perceived handicapping effects due to a vestibular disorders ^69^. Criterion validity of this questionnaire has been confirmed within the TBI population, showing excellent correlation between the DHI and objective visual and balance measures ^70, 71^. The DHI has been reported to have content validity among TBI patients ^72, 73^. The *Neurobehavioral Symptom Inventory* (NSI) is a 22-item self-report questionnaire recommended for standardization by the Office of Outcomes and Assessments and the Defense and Veterans Brain Injury Center ^74^. The NSI was developed to assess a wide range of symptoms in patients presenting with post concussive syndrome” ^74^. Widely used by the DoD, the validity of the NSI has been confirmed through studies demonstrating internal consistency and scores correlating with clinician-confirmed TBI ^75^. The *Quality of Life after Brain Injury scale (QOLIBRI)* is a questionnaire with 37 items addressing 6 dimensions of quality of life; cognition, self, daily life and autonomy, social relationships, physical problems and feeling bothered by emotions ^76^. The QOLIBRI has established test-retest reliability and internal consistency in assessing quality of life across cultures in patients with TBI ^76^. The *Concussion Clinical Profile Screen (CP Screen)* ^36^ is a 29-item scale that is organized around concussion clinical profiles: 1) anxiety/mood (5 items), 2) cognitive/fatigue (3 items), 3) migraine (5 items), 4) ocular (5 items), and 5) vestibular (5 items); and 2 modifiers: 1) sleep (4 items), and 2) neck (cervical) (2 items). Participants respond to each item on a 0 (none) to 3 (severe) Likert-type scale. The CP Screen provides a total symptom score ranging from 0 to 87 and individual subscale scores ^36^.

*Qualitative questions:* We will incorporate qualitative feedback both 1) from the physical therapist’s impression of using the biofeedback system with the participants and 2) from the participants at the end of the rehabilitation. We held a focus group with several stakeholders to solicit a concise set of questions to include. Stakeholders included mTBI research experts, research and clinical physical therapists, a consultant with lived experience, and a psychometrician/methodologist. While we plan to use the PGIC as an outcome measure, we will also ask the participants with mTBI the following, *“Please describe how each of the following aspects of change (activity, symptoms, emotions and overall) factored into your rating on the Global Impression of Change scale.”*

We will ask the participant to discuss their return to work/study and their return to activity. For return to work/study, we will use a scale adapted from Moore et al. and van der Naalt et al; (0=previous work/study resumed or full return to duty, 1= resumed with lower demands or part time or on limited duty/profile, 2= not resumed or different work on a significantly lower level or reassignment, 3=unemployed/not studying or recommended for medical board) ^77, 78^. The participant will then be asked to elaborate on facilitators and barriers in achieving this level of return to work/study. For return to activity, we will ask about the level of intensity and types of activities they are currently participating in compared to previous (prior to the mTBI) using a scale adapted from Moore et al; (0 = participation in previous physical/recreational activities resumed, 1 = previous physical/recreational activities resumes with lower demands, 2 = not resumed or different activities on a significantly lower level, 3 = inactive/no participation in physical/recreational activities) ^77^. The participant will then be asked to elaborate on facilitators and barriers in achieving this level of return to activity. We will ask the physical therapist at each site one open-ended question: *“Would you continue to use this feedback system after the study is over. Please discuss why or why not.”*

*Table 4. Outcome measures*

| **Patient-reported (subjective)** | **Clinical Assessment** | **Instrumented Assessment** |
| --- | --- | --- |
| Patient Global Impression of Change (PGIC-**Primary)**  Dizziness Handicap Inventory (DHI)  Neurobehavioral Symptoms Inventory (NSI)  Quality of Life after Brain Injury (QOLIBRI)  Concussion Clinical Profile Screen (CP Screen) (used as total symptom score (Aim I) and for profile (Aim II and III)  Qualitative *open-ended* for mTBI participants and physical therapists | *Balance:*  Balance Error Scoring System (BESS)  modified Central Test of Sensory Integration on Balance (mCTSIB)  *Gait:*  Functional Gait Assessment (FGA)  2-min walk  *Turns:*  Custom Clinical Turning Course (CCTC)  *Vestibular/ocular-motor:*  Vestibular/Ocular Motor Screening (VOMS)  Clinical Dynamic Visual Acuity | *Balance:*  Instrumented BESS  Instrumented mCTSIB  *Gait:*  Instrumented FGA  Instrumented2-min walk  *Turns:*  Instrumented CCTC  *Vestibular/ocular-motor:*  Laboratory measures including: Dix-Hallpike, smooth pursuit, saccades, vHIT |
| **Covariates for Aim I**  Age, gender, previous mTBI history  Post-traumatic stress disorder (PTSD Checklist) | | |

***Clinical Assessment:*** The *Balance Error Scoring System (BESS)* assesses balance by asking participants to stand in progressively challenging conditions (feet together, single limb stance and tandem) both on firm and foam surfaces ^39^. All items are performed with eyes closed. A rater evaluates the number of errors committed by the participant, such as opening their eyes or removing their hands from their hips during the first 20 seconds of the trial. The modified Clinical Test for Sensory Integration on Balance (mCTSIB) ^79^ assesses the sensory contributions to balance. In this test, participants stand with feet together in 4 conditions; 1) eyes open on a firm surface, 2) eyes closed on a firm surface, 3) eyes open on foam surface, and 4) eyes closed on a foam surface. Participants are clinician-rated on stability based on the ability to maintain balance for 30 seconds in each condition. The *Functional Gait Assessment (FGA)* evaluates a patient’s ability to adapt their balance while walking and undertaking a series of additional tasks such as turning their head, changing speeds, and stepping over an obstacle. The FGA is a tool that can differentiate gait differences between healthy people and those with mTBI (AUC = 0.78) ^80^. The patient’s performance on the test is rated by a clinician on a scale of 0 to 30 with higher scores indicating better performance. *Two-minute walk* ^81^ is a measurement of endurance that measures walking distance over 2 minutes. The *Custom Clinical Turning Course (CCTC*) is a test of mobility that evaluates a person’s ability to walk a prescribed path involving multiple turns of various angles ^82^. Individuals walk around the course at their preferred normal walking speed. The course is marked with arrows taped on the ground indicating turns of 45°, 90°, and 135°. Participants complete a minimum of 6 laps, and the time is recorded via a stopwatch. The average time to complete each lap is recorded, with faster lap times indicating better performance. The *Vestibular Ocular Motor Screening (VOMS*) was designed to examine how extra-ocular and head movements (e.g., saccades, smooth pursuits) provoke symptoms in individuals post-mTBI ^83^. Each eye or head movement task on the VOMS is scored from 0-10 with higher scores indicating more provocation of symptoms. The VOMS assessment examines how smooth pursuit, horizontal saccades, vertical saccades, convergence, horizontal VOR, vertical VOR, and visual motion sensitivity, affect headache, dizziness, nausea, and fogginess symptoms. At baseline and after each condition, the symptoms are scored from 0-10, with higher scores indicating more provocation of symptoms. *Dynamic Visual Acuity (DVA) ^84^* assesses gaze stability during yaw plane head rotations relative to head-stationary visual acuity. First, static visual acuity is assessed with the head stationary and the patient looking straight ahead while seated 4 meters from an Early Treatment of Diabetic Retinopathy Study (ETDRS) visual acuity chart. The patient reads the lowest line recognizable and keeps reading until they can no longer identify all the letters on a given line. The examiner notes the last line where all letters were correctly identified and the total number of letters correctly identified. For the dynamic portion of the test, the examiner stands behind the research participant and holds their head with both hands. Starting at least 5 lines above the last line read during the static acuity testing, the examiner rotates the participants head in an arc of 20 degrees on either side of midline in time with a metronome set to 240 beats per minute, to ensure that the VOR is prioritized to stabilize vision. Again, the participant reads the letters on the lowest line of the eye chart possible and the examiner notes the last line where all letters were correctly identified. The number of line difference between static and dynamic acuity tests will be compared and a loss of three or more lines of visual acuity relative to one’s static visual acuity will be regarded as clinically meaningful and suggestive of gaze stabilization difficulty^85^.

***Instrumented Assessment:*** Instrumented assessments of balance and gait will use synchronized inertial sensors (Opals by APDM; Figure 13). The Opals have 3D accelerometers (range ±6 g), gyroscopes (range ± 2000°/s), and magnetometers (range ±6 gauss). Opal sensors will be applied to the head, sternum, lumbar spine and feet with elastic Velcro bands. Inertial data collected at 128 Hz will be synchronized and wirelessly transferred to a laptop for automatic generation of metrics with raw data stored for further analysis. The Opals are designed for easy use; they require no switches or configuration by the user. The instrumented system uses Mobility Lab software to enable sensor configuration, recording, real-time visualization, and data management. Mobility Lab provides sensitive, valid, and reliable outcome measures of gait and balance ^46, 67, 86^. Study data is exported from the Mobility Lab desktop software to the Mobility Exchange server, using an HTTPS channel employing a 2048-bit RSA encryption. Data integrity checks happen up-front, during the data collection and analysis process. When using the in-clinic data collection mode, users are alerted about integrity issues immediately and provided the opportunity to re-record the test. The instrumented BESS ^87^, mCTSIB ^43^, FGA, 2 minute walk and CCTC ^88^ will occur simultaneously with the clinical test (see above).


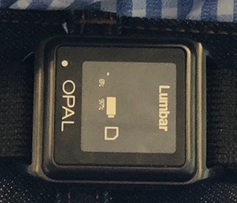


Figure 13. Watch size Opal sensor

*Instrumented BESS* – Postural sway measured by an Opal sensor on the waist will be evaluated simultaneously with the BESS described above ^42, 87^. Postural sway will be automatically quantified in the ML direction using APDM software during each stance condition by calculating the root-mean-square (RMS) around the mean acceleration, a metric representing sway dispersion. Our primary measure for this test will be the averaged ML RMS. Our previous research identified this stance condition and metric as the best for identifying individuals with mTBI ^87^. Other temporal and spatial metrics such as sway area, velocity, frequency and jerkiness will be simultaneously collected for secondary analysis. *Instrumented mCTSIB* – Postural sway will also be quantified using an Opal sensor during the clinical mCTSIB. The same outcomes of sway will be obtained for the Instrumented mCTSIB as for the Instrumented BESS above. *Instrumented FGA -* Participants will complete all of the walking tasks that are components of the FGA. Based on our previous research, we are particularly interested in 4 items: Gait Level Surface (FGA-1); Gait with Horizontal Head Turns (FGA-3); Gait with Vertical Head Turns (FGA-4); and Gait with Eyes Closed (FGA-8). Additionally, the head turn kinematics will be quantified during FGA-3 and FGA-4) ^89^. Peak angular displacements and velocities of the head and trunk will be extracted from the angular velocity data. Head movement variability (e.g., RMS of head acceleration) during the FGA-1 and FGA-8 will also be calculated using custom MATLAB algorithms. *Instrumented Gait* – Participants will walk at their self-selected pace for 2 minutes between two lines. Spatiotemporal gait metrics will be obtained from the APDM Mobility Lab software ^67^ Measures will be used to identify gait domains associated with mTBI, including Variability, Rhythm, Pace, and Turning ^90^.

*Instrumented CCTC* – Turning velocity and segmental coordination will be quantified using Opal inertial sensors located on the head, sternum, and lumbar regions as participants complete the CCTC described above (Figure 14). The peak axial rotation (i.e., yaw) angular velocity and segmental coordination are extracted for each turn (45°, 90°, 135°) using custom MATLAB algorithms developed by our research team. Using these methods, our previous studies have shown that the peak angular velocity is sensitive to mTBI ^91^ and can be equivalently collected across multiple sites ^88^. Slower turning velocities indicate more impaired function ^88^.


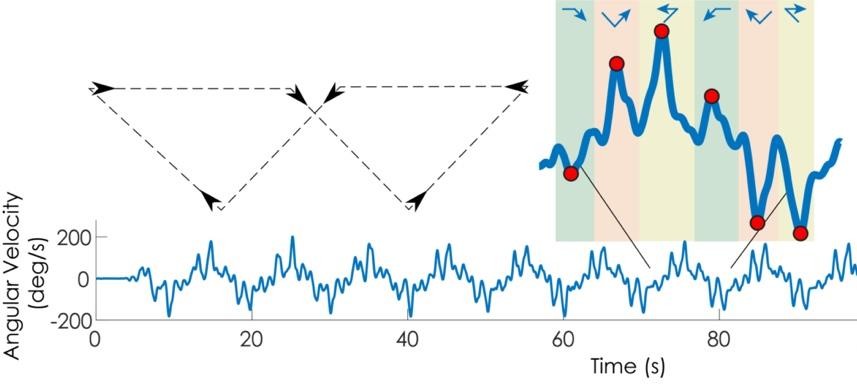


Figure 14. Custom Clinical Turning Course (top left) shown with angular velocity timeseries data (bottom). The timeseries depicts 9 laps, and data from one lap is enlarged (top right) to illustrate the turns at each angle. Different angles of turns are shaded with different colored bands. Turns of 45 degrees are shaded green, turns of 90 degrees are shaded red, and turns of 135 degrees are shaded yellow

*Instrumented Vestibular and Ocular-motor measures* – Commercial (Interacoustics VisualEyes 525, Neurolign I-Portal NOTC and VEST, Natus Otometrics ICS Chartr 200, and Natus Otometrics ICS Impulse) systems will be used to assess vestibular and ocular-motor function based on quantitative analysis of infrared video eye movement recordings. Vestibular function evaluation will include Dix-Hallpike and Roll tests for the identification of benign paroxysmal positional vertigo (BPPV), video head impulse tests (vHIT) for the detection of reduced or asymmetric vestibular function, and measurement of spontaneous and positional nystagmus in the dark. Ocular-motor function assessments will include horizontal (0.3 and 0.75 Hz) and vertical (0.3 Hz) smooth pursuit eye tracking of sinusoidally moving targets, and horizontal and vertical saccades to visual targets appearing in random locations. These commercial systems analyze recorded eye movements that provide quantitative measures of performance (examples: vHIT gain, comparing the ratio of evoked eye velocity to head velocity and vHIT asymmetry, comparing gains for head movements to the right versus the left; smooth pursuit eye velocity gains and asymmetry; saccade onset latency and accuracy). Established clinical norms for these tests will be used to classify participants into normal versus abnormal vestibular and ocular-motor function categories.

*Potential covariates and confounders*, in agreement with the CDE recommendations, will be measured. These measures are CDE’s for both the NINDS and the Federal Interagency Traumatic Brain Injury Research (FITBIR):1) Age, gender, significant medical history including previous mTBI history (prior mTBIs, loss or alteration of consciousness, length of post-traumatic amnesia), 2) Post- traumatic stress disorders (PTSD) using the PTSD Checklist for Civilians or PTSD Checklist for Veterans ^92, 93^

*Statistical Analysis Plan.* The PGIC, our primary outcome, is an ordinal variable; participants rate their global assessment for change on a scale from 1 to 7. Therefore, to test whether multidimensional real-time biofeedback has better outcomes than standard care, we will use this variable and will first conduct a Mann-Whitney U test to assess statistically significant group differences in PGIC scores. To consider the influence of relevant covariates (e.g., age, gender, PTSD, etc.), subsequent analyses will use ordinal logistic regression. This will test for group differences while controlling for covariates. To account for multiple comparisons, we will set alpha so that familywise error does not exceed 0.05. *Secondary Outcomes: Patient reported symptoms, clinical object, and instrumented objective measures* – We will measure several secondary outcomes from patient reported outcomes, clinical assessment, and instrumented assessment at baseline and post-rehabilitation (see Table 4 for a complete list of measures and covariates). We will calculate a change score between the pre- and post-test scores for each participant. We will assess each variable for violations of normality. If normality is violated, we will perform a data transformation (e.g., log transformation). For outcomes that are approximately normal, we will test for group differences in change for each outcome using a General Linear Model (GLM) approach to Analysis of Covariance (ANCOVA). This will allow us to test for group differences while controlling for the possible effects of the covariates. The GLM approach has less stringent assumptions than ANCOVA regarding equal sample size and variance within groups and will allow a more robust assessment of group differences. If outcome variables violate normality and are not corrected with a data transformation, we will use the Kruskal-Wallis nonparametric approach to test for group differences.

*Qualitative Outcomes.* Participant responses will help contextualize the quantitative measures and provide a more nuanced understanding of how patients regard their response to therapy. To minimize bias, researchers administering surveys to participants will be trained on how to ask the questions and use pre-prescribed prompts to elicit participants’ responses. Responses will be recorded and we will transcribe participants’ responses to each of the open-ended questions. Our team includes a statistician that has experience in qualitative data analysis including analyzing data from focus groups from transcribed oral responses. From these transcriptions, she will identify themes that we will use to further our interpretation of the usefulness of the multidimensional real-time biofeedback system and to help understand the relevance of multiple measures of recovery.

## *Potential Problems*. *Recruitment* – We may have difficulty recruiting enough people but this is unlikely given our previous success with similar recruitment criteria at all sites. All sites have established recruitment methods already in place. It is also possible we will enroll a subject that does not have either a vestibular and/or ocular-motor deficit, but we think this will be unlikely as we will only enroll people who have some symptoms of V/O complaints (See *Justification* above for V/O cut-off scores). Further, we will prescreen participants using the CP Screen to make sure we have equal number of people with more severe V/O subtype compared to minimal V/O deficits. *Attrition* – We may have high numbers of people who drop out of the study but we factored in an estimated 25% drop-out rate so we don’t anticipate this to be a problem. *Technical* – We may experience technical difficulties with wearable sensor-based feedback system. All proposed sites have some experience using wearable technology and have had minimal technical issues with previous studies. With our expertise in embedded system technology, we will be able to handle technical issues as they arise. We have already solved many problems in the development phase such as minimizing the delay and providing a system with low latency and optimized energy usage. The sensing/controlling unit was designed to have the speed needed to detect and communicate objective measures in the shortest possible time.

## Aim II. Responsiveness to rehabilitation; objective measures to identify V/O subtype. To explore a) rehabilitation efficacy across severity of V/O deficits, using multiple measures of V/O function and b) the strength of the relationship between subjective and objective measures of V/O function.

*Rationale.* While objective measures have been recently highlighted as a critical component of ass**ess**ment after mTBI, self-reported measures continue to drive medical referrals, clinical subtyping and plan of care. It is documented that V/O subtypes are common with approximately 35% of patients categorized with V/O as the primary subtype in a retrospective chart review ^94^. We believe that using both subjective and objective measures of V/O function will result in a more accurate classification of subtypes, leading to the better identification of V/O subtypes, creating more appropriate referrals and precision medicine.

*Design and Participants*. This is an observational study where we will explore responsiveness to rehabilitation and relationships across multiple measures of V/O function including Patient-reported, Clinical Assessment and Instrumented Assessment. We will use the data acquired from participants in Aim I to explore responsiveness to rehabilitation and to explore the relationship among measures of V/O function.

*Power and sample size considerations for Aim II.* Because this is an exploratory Aim, we will use estimates from the data to inform power and sample size calculations for future studies*.* Therefore, we will use data from all participants from Aim I.

*Statistical analysis plan.* In this exploratory aim, we will: 1) assess V/O HI versus V/O LO participants’ responsiveness to rehabilitation and 2) explore the relationship between subjective and objective measures of V/O domains. Responsiveness to rehabilitation will be determined by the participants score on the PGIC after rehabilitation. PGIC score will be dichotomized by “responder” or “non-responder”^51^. We will classify participants who score 6 or above on the PGIC as “responders” and those who score 5 or below as “non-responders” (Figure 15). First, we will examine the distribution of PGIC scores and the proportion of responders vs. non-responders in V/O HI group and V/O LO group. Then we will conduct a logistic regression on responder vs. non-responder as the binary outcome to assess differences between V/O HI group and V/O LO group. For the next step, we will assess the PGIC as an ordinal outcome variable and test group differences in responsiveness using ordinal logistic regression. Both regression models will allow us to control for covariates in the models. We will use the data to obtain estimates to inform future studies and power calculations. Finally, to explore the strength of associations between the Patient-reported, Clinical, and Instrumented V/O measures (see Table 4), we will measure the associations using Pearson correlation coefficients for continuous variables and Spearman correlation coefficients for ordinal variables.


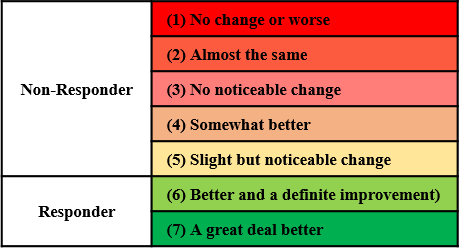


Figure 15. Binary cut off scores to define responsiveness to rehabilitation on the PGIC.

*Outcomes.* The outcomes for Aim II are the same outcomes measured for Aim I (see Table 4).

*Interpretation.* If we find that the people with high V/O deficits are more responsive to an evidence-based, multimodal, 6-week physical therapy intervention for people with mTBI compared to those with milder V/O deficits (that may include other subtypes such as cognitive, headache/migraine or anxiety/mood), we will conclude that it is essential that subtype classification occur routinely, prior to initiating physical therapy. If we find that there are only small or moderate associations between subjective and objective measures of V/O function, we will conclude that mTBI subtyping should include more than just subjective symptom reporting and that objective measures are essential for routine clinical assessment prior to referrals and targeted treatments.

*Potential problems*. We anticipate varied responses for the PGIC. In the event that responses are homogeneous, this will limit conclusions that we can draw on responders versus non-responders. However, we also have numerous secondary measures across levels including subjective, clinical, and instrumented measures that will inform and motivate future studies.

**Aim III: Daily life mobility in people with V/O subtype.** To: a) determine if daily life mobility (quality of gait and turning) is impacted differently in people with high versus low V/O deficits, b) calculate healthy normative data for daily life mobility measures in active duty military service members.

*Rationale*. We recently published a study demonstrating that quality (but not quantity) of daily life mobility is different in people with chronic mTBI ^48^ (Figure 4) and the proposed study will now explore whether people with more severe V/O symptoms have worse quality of daily mobility. We are particularly interested in continuous monitoring as an outcome measure since there are very few participation level outcomes used according to the International Classification of Function, Disability and Health (ICF) model, a model that provides a standard framework for assessing a person’s health and health-related state ^95^. Several reports identify the concern that studies use minimal participation level assessments and currently the majority of participation level outcomes are self-reported measures. Understanding changes at the participation level is vital to know if and how injury and/or rehabilitation effects daily life ^95-97^.

We will also obtain healthy normative values for military active duty service members since this population may have higher and more variable activity levels than civilians. Specifically, physical fitness and exercise training are emphasized in the armed forces to meet the physical demands of the job, while such demands are not as widely expected within the careers of the civilian population ^98^. Further, military personnel are reported to have more regimented engagement in physical activity, excel in meeting weight standards and have access to more formalized exercise training which many civilians are not offered in places of employment ^99-101^. The 40 healthy control military service members will provide daily life mobility control data that will be useful in assessing global function of service members post-mTBI and ultimately could be useful for return to duty decisions.

*Participants.* For Aim III, we will enroll the first 25 people who classify as V/O HI and the first 25 people who rate as V/O LO, as measured in the CP Screen (See *justification* above for cut-off scores). For the 40 healthy active duty military personnel, service members working at FSH will be recruited primarily from the U.S. Army Medical Center of Excellence. Participants must be active duty military service members with no recent (<3 year) history of mTBI and no residual symptoms from any other previous mTBIs to be enrolled.

*Power and sample size consideration.* Preliminary pilot data were collected from eight participants on objective daily life mobility metrics. In the pilot study, the participants were categorized as having either moderate to severe V/O deficits (n=3) or minimal V/O deficits (n=5) based on the Concussion Symptom Subtype Inventory (CSSI)^37^. For the purpose of power analysis for Aim III, three quality metrics including turn angle (deg), turn angle variability (CV), and turn peak velocity (deg/s) were assessed between two groups to obtain the parameters. From our preliminary data, the estimated mean difference between two groups and common standard deviation (SD) of turn angle, turn angle variability and turn peak velocity are 4.48(±4.14), 0.018(±0.016), and 7.95(±4.47), respectively, which yielded very strong effect sizes (1.1, 1.1 and 1.8). Therefore, we conservatively determine sample size calculation with the effect size of 0.90 when comparing two groups. To gain at least 80% power to detect the difference, we need at least 21 subjects for each group, at a level of significance alpha = 0.05. Therefore, we aim for collecting measurements from at least 21 subjects per group (N=42). Given an estimated completion rate larger than 85%, we plan to enroll 25 subjects per each group (N=50).

*Procedure.* Participants will wear 3 sensors, one on each foot (embedded in neoprene instrumented socks, Figure 3) and one on the waist (on elastic belt) to passively quantify gait and turning during 7 days of daily life. The 50 participants (25 from OHSU and 25 from UU) will undergo 7 days of continuous, passive monitoring and the 40 active-duty military service members will also undergo 1 week of continuous monitoring. In the morning, participants will don the instrumented socks (Figure 3) and the belt-worn sensor. The sensors will be worn all day and then inserted into a docking station to charge each night. The cutting-edge technological approach will allow us to develop sensitive, functional test batteries for assessing and treating mobility disorders for use in future clinical trials and practice.

*Daily life mobility measures;* The measures of mobility during daily life will be calculated with either proprietary algorithms from *APDM Wearable Technologies* or our Balance Disorders Laboratory algorithms, all of which have been validated ^45-47, 67^. Measures are calculated by combining the 3 axes of linear acceleration with 3 axes of angular velocity and the magnetometer, all sampled at 128 Hz to obtain motion and orientation of the feet and body in space. The APDM wearable sensors have a unique, patent-protected, wireless synchronization of the sensors that allows precise temporal binding of data across sensors. Our partnership with *APDM Wearable Technologies* resulted in a series of NIH-SBIR grants focused on developing and testing these novel instrumented socks, consisting of inertial sensors on top of the feet connected to a long-lasting battery on the lower leg ^47^. Data are automatically stored in the internal memory of the sensors and automatically uploaded to a secure data server (using HIPAA compliant AWS services) when docked to charge at night. Incremental backups of the data on all Mobility Exchange instances are performed daily. Redundant copies of these backups are transferred daily to a separate AWS region, managed by AWS services. In parallel to this innovative hardware, we have also developed and validated algorithms to analyze turning and straight-ahead gait during daily life ^45, 67^. Specifically, for turning we validated inertial sensor algorithms to measure turning quality for 45-360 degree angles performed at normal, fast and slow gait speeds during continuous walking tasks compared to gold-standard, motion analysis and for daily living compared to video-data from a waist-mounted, GoPro mini-camera aimed at participants’ feet during normal activities in the home ^45^. The algorithms showed a sensitivity of 0.90 and a specificity of 0.75 for detecting the start and end of each spontaneous turn compared to motion analysis. Sensitivity and specificity of the algorithm compared to video-raters for home measurement was 0.75 and 0.77^45^. Our studies not only demonstrated the feasibility of the method for continuous monitoring of mobility up to 14 hours per day, but also validated the ability to use the same gait and turning algorithms during daily life as used in the laboratory, after identifying walking bouts and standing and turning periods ^47^. Further details on metrics extractions are described in previous publications ^45, 46, 67^.

*Outcomes.* We will focus on a subset of daily life passive monitoring measures (Table 5), based on our published results and pilot data ^48^. Out of 43 different measures of gait and turning (plus their variability as SD) obtained from 7 days of daily life passive monitoring, several turning measures and their variability were consistently different between people with mTBI and healthy control participants ^48,53^. Specifically, people with mTBI avoided large turn angles, turned slower and had less variability throughout their daily mobility. Our pilot data suggested that similar measures were different in people with objective deficits in V/O domains, compared to those without (Figure 10). We will use the same measures outlined in Table 5 to test our hypothesis that quality of daily life mobility is worse in people with V/O HI deficits. Additionally, as the mobility measures listed in Table 5 are those that differ the most between controls and people with mTBI, we expect that rehabilitation-related changes will be revealed by these measures.

*Table 5: Outcome measures from continuous monitoring*

| **Quality Turning Measures** | **Quantity Activity Measures** |
| --- | --- |
| Turn Angle (°) and variability  **(Primary**) | Activity Rate |
| Turn Duration (seconds) and its variability | Number of steps per day |
| Peak turn velocity (°/second) and its variability | Number of turns per hour |
| ***Covariates for Aim III:***  Age, gender, previous mTBI history  Post-Traumatic Stress Disorder (PTSD Checklist  The International Physical Activity Questionnaire (IPAQ), Life Space Assessment Questionnaire | |

Covariates are described under Aim I outcomes and for Aim III we will add activity measures including the International Physical Activity Questionnaire (IPAQ), a valid and reliable self-report on activity level ^102^ and the Life Space Assessment questionnaire, a reliable self-report on the area in which a person lives ^103^ .

*Statistical Analysis Plan.* People will be grouped using the CP Screen according to their level of severity of V/O deficits (HI or LO V/O deficits) to test the hypotheses for Aim III. Prior to detailed statistical modeling, we will compute quality metrics summarized over the duration devices were worn by each participant (for those that wore the devices for at least 5 out of 7 days and at least 10 hours/day). Specifically, we will provide both statistical and graphical evaluation for the distributions of three quality metrics: turn angle, turn angle variability, and turn peak velocity, and then transform the values if necessary to better meet model assumptions. Then we will examine the distribution of the three quality metrics in each group. The descriptive statistics of quality metrics for each group will be reported as mean (SD). To test whether daily life mobility measures differ between V/O HI and V/O LO groups, we will test on the quality metrics for group differences using an independent sample t-test. In addition, we will conduct subsequent analyses to compare quality metrics between two groups accounting for covariates (Table 5) using the multivariate generalized linear modeling (GLM) approach. The model will be constructed for each quality metric (outcome) separately. The group estimates will be assessed with and without the co-existing covariates to explore potential confounding factors, which allows us to test for group differences while controlling for the possible effects of other covariates. In addition, for turn peak velocity variability and quantity activity measures, we will also examine the distributions using the same method and conduct two independent sample t-test between two groups for the completeness of the analysis.

To summarize the healthy military normative group, we will similarly collect data from instrumented socks worn over a 7-day period. We have normative data already collected on healthy civilians, however, there is evidence that suggests that people in the military might have different activity levels compared to the general population ^101^. Therefore, we will use the data we gather from healthy military participants to calculate normative values across all metrics. We will examine the distribution of the quality metrics and provide descriptive statistics including means, standard deviations, median and 25^th^, 75^th^ percentiles. Additionally, we will use histograms and Shapiro-Wilk’s tests to check the normality of the data. This will provide preliminary data for our next studies exploring daily life mobility in those cleared to return to duty after mTBI.

*Interpretation.* If we find that turning quality, measured during daily life, looks different in people with high versus low V/O symptoms, this would support adding this as an outcome measure to help direct rehabilitation and track recovery. A future study should explore if service members cleared to return to duty post-mTBI have the same level of daily life mobility as healthy service members (a value established in Aim III). If they do not, perhaps return to duty decisions were made too soon or rehabilitation was incomplete.

*Potential problems. Technical:* It is possible that the Opal sensors will fail to synchronize after charging or that subjects will wear them inappropriately. However, our experience with over 300 older people with multiple diagnoses (some with mild cognitive impairment) wearing Opals for 7 days tells us these types of technical issues will be quite limited. Nevertheless, the research assistant will call participants the day after issuing the system to mitigate technical issues. Nightly data upload and data quality checks will enable early detection of these issues. *Compliance:* We may find that people do not wear the instrumented socks for enough hours/day or days/week. It is unlikely, but possible that the participants may be unable to complete the 7 days of recording. If this occurs, only the completed period will be used in the performance assessment. If the period is shorter than 5 days, we will then recruit additional people to reach our target enrollments. Compliance data from our previous studies showed that people rated wearing our instrumented socks comfortable for seven days during daily activities*. Subtype categorization*: In the event that we don’t have enough representation of participants with mild V/O deficits, we will revisit categorization criteria or recruit more mild V/O patients.

**Screening Procedures:** The research assistant will ask you prescreening questions over the phone to determine eligibility for the study. If you appear to meet all inclusion and exclusion criteria, as listed above, the research assistant will schedule an initial appointment. During this session, the research assistant will ask additional screening questions, including pertinent medical history, after consent is obtained.

**Risks/Benefits Assessment:** These are safety measures to minimize and/or eliminate risks to human subjects and personnel.

- Falls: The proposed team of researchers has tested over 500 participants using similar tests of postural control in various projects at OHSU without significant injury to any participant. A trained and experienced assistant will ‘spot’ you during all balance testing to be ready to catch you if needed. In addition, if you have especially poor balance, a safety belt can be placed on you before balance testing to make it easier to spot during clinical testing. It is therefore highly unlikely that you will experience a loss of balance or be injured during testing.
- Exacerbation of symptoms: If symptoms of dizziness, headache or nausea are exacerbated, the session will not proceed until your symptoms have decreased. Additionally, we will have designated rest periods during the protocol for safety.
- Loss of confidentiality: The computer that will store the project database will be protected by current network security. An institutional computer account and password will be required to access the computer storing the recruitment database and the images. Data will be maintained on secure, encrypted servers maintained by OHSU.
- Emotional anxiety: Should emotional anxiety result from testing or questionnaires, we will inform you that the testing does not reveal information about the severity of your problems, as this is not the purpose of the study. We will encourage you to discuss any balance problems with your treating physician and physical therapists.
- Physical injury: There is a low risk of injury during or after a testing session. The risk is reduced by use of well-trained assistants and by the mild nature of the gait, balance and vestibular testing.
- Loss of privacy: You will be assigned a coded identification number and this number will be used to keep track of your data. Only individuals who are specifically authorized to view private health information by the principal investigator will have access to information that could be used to identify you. Thus, the risk to you in this research is in the categories of unlikely physical risks and potential loss of information privacy.
- Wearable sensor system: The wearable sensors worn on the body are commonly use in other laboratory studies assessing balance and gait. There is no identifiable information stored on these sensors. These sensors are non-invasive but may cause minimal discomfort similar to wearing a headband, wristwatch or belt.
- Biofeedback sensor system: These sensors used for biofeedback are the same as the sensors in the wearable sensor system. There is no identifiable information stored on theses sensors. These sensors are non-invasive and may cause minimal discomfort similar to wearing a headband, wristwatch or belt.

**Risk Management and emergency response:**

Procedures in this study were specifically designed to minimize risks to the participants. However, Dr. King (overseeing principal investigator) or her designee will review data checks monthly to ensure routine evaluation of events, to determine any potential unanticipated problems that may involve risks to subjects or others, and the appropriate action as a result of such events. Study personnel will be responsible for reporting adverse events as they occur to Dr. King, who will notify the OHSU IRB of the occurrence within 5 days of the event. Serious adverse events (life-threatening or disabling and requiring medical attention) that may occur during this study, such as death and cardiovascular events, Dr. King, and all other investigators, will be notified immediately and will submit a full written report to the IRB within 24 hours. While these events are extremely rare in persons engaging in low or moderate activity, it is possible that a person with previously undisclosed cardiovascular disease may experience a cardiovascular event or death during activity. In the event this occurs, study personnel will take the appropriate, necessary measures. However, this federally funded study will not have the ability to provide compensation for research-related injury. Emergency treatment may be available but subjects and/or their insurance company will be charged for this treatment.  Events such as dropouts and protocol deviations will be reported to Dr. King and they will be reported to the IRB during annual continuing reviews.

**Potential benefits:** You may or may not personally benefit from being in this study. However, by serving as a subject, you may help us learn how to benefit patients in the future. You will receive $75 for the first testing session and $100 for post intervention assessment. If you are asked to wear the sensors at home for 7 days, you will receive an additional $10/day totaling $70.

**Data Management**

**Identifiers:** You will be assigned a coded study identification (ID) number generated after written informed consent has been obtained.  The your name and unique ID number will be recorded in an electronic file, stored on a password-protected secure drive. Research records will be stored in a confidential manner so as to protect the confidentiality of subject information. Data collection forms will be labeled using unique study ID numbers and will not have personal identifiers.

**Confidentiality:** To ensure and protect your privacy and to maintain confidentiality of the data, the following activities for data and safety monitoring will be in place for the study: 1) A detailed plan will be approved through a multi-level Institutional Review Board (IRB) approval process (see Study Personnel and Organization: Study Management Plan) before recruitment begins, 2) Data will be coded upon collection with the key to the code held separately from the data and accessible only to IRB approved study personnel who need to know. Subject information and study data will be stored in locked cabinets (if paper) at each site and behind a secure Oregon Health & Science University (OHSU) firewall on network drives requiring password authentication. The representatives of the US Army Medical Research and Material Command (USAMRMC) are eligible to review study records. The persons who are authorized to use and disclose the data from this study are the approved investigators, other research professionals at each site who are participating in the conduct of this research protocol, and the OHSU IRB. The IRB at each site is authorized to use and disclose data relating to participants tested that that site. All data will be safeguarded in accordance with Health Insurance Portability and Accountability Act (HIPAA). The knowledge of any pending compliance inspection/visit by the USAMRMC, or other government agency concerning clinical investigation or research, the issuance of Inspection Reports, warning letters, or actions taken by any Regulatory Agencies, including legal or medical actions, and any instances of serious or continuing noncompliance with the regulations or requirements will be reported immediately to the site-specific IRB, to OHSU, and to the Department of Defense and/or USAMRMC. Representatives from USAMRMC are eligible to review study records upon request. Data collected in this study do not include any sensitive information, such as history of communicable diseases that are required to be reported to state or local authorities.

**Sharing study results:** The results generated in this study are experimental in nature and will not be shared with study subjects.

**Laboratory Evaluations:** Not applicable; no specimens will be collected for study purposes.

**Data capture, verification and disposition:** The multicenter protocol will rely on a web-based system  of data input into a central Research Electronic Data Capture system (REDCap). REDCap is a secure electronic database administered by OHSU and maintained on secure servers with limited access. The system will feature multi-tiered security-protected access and will conform to HIPAA security policies. Stored data will be backed up daily. The de-identified Mobility Lab data will be collected on a password-protected and data-encrypted laptop computer and uploaded after each test session to an OHSU secure server. Authenticated investigators will have access to the dataset from any secure Internet access point. Computers that will store the project database will be protected by current network security behind an OHSU firewall. All investigators and key study personnel will be provided with an institutional OHSU account to access REDCap. An institutional computer account and password will be required to access the computer storing the recruitment database and the images. In addition, the REDCap database is password protected, and only project personnel approved by Dr. King will be given access.

Data will be gathered from questionnaires and instrumented assessments, and entered into the REDCap web-based data entry portal by research assistants at each site. The investigator or coordinator at each site will subsequently confirm the accuracy of data entry for every third participant by comparing electronic data with the original hard copy data. Once sites have completed their data checks then OHSU’s study coordinator will investigate any discrepancies or incomplete data entries. All forms will be locked by OHSU’s study coordinator once data verification is complete.

The feedback system utilizes wearable inertial sensors (Opals) for precise quantification of motion. The Opals log kinematic data to on-board memory, which can be transmitted to a PC either wirelessly or via a USB adapter. When used in the clinic, the system uses a Docking Station to recharge the Opal's battery and transfer the raw sensor data logged to a computer and an Access Point to enable wireless streaming to a computer. The system also includes a cloud-based Clinical Data Management System called Mobility Exchange, which is

designed to help execute multi-site clinical trials employing the software for data collection. The primary functionality of the Mobility Exchange server is to support the aggregation of data collected from multiple systems, support the centralized administration of a clinical study, and to implement technical controls to satisfy regulatory requirements and guidance.

Mobility Exchange uses HIPAA-compliant Amazon Web Services (AWS) services, and it manages user access, tracks changes in an audit log, ensures valid data uploads, and stores each site’s data (raw and analyzed) in a central repository. Incremental backups of the data on all Mobility Exchange instances will be performed daily. Redundant copies of these backups are transferred daily to a separate AWS region. These are managed by AWS services.

Hard-copy research records and consent forms will be stored in a locked file cabinet within a locked, secure office. Hard-copy and electronic research records will be kept for seven years, in compliance with HIPAA, or one year after publication, whichever is longer. All identifiers will be removed at the completion of the study and it will not be possible to link the data to individuals after that time.

**ACCESS TO YOUR TEST RESULTS**: The results of research tests will not be made available to you because the research is still in an early phase and the reliability of the results is unknown. We are asking you to provide your information for a data bank, also called a repository. These samples will be stored indefinitely and may be used and disclosed in the future for research.

|  | **Pre-Rehab Testing** | **7 Days Home Monitoring with SmartSox** | **Intervention (with or without biofeedback)** | **Post-Rehab Testing** |
| --- | --- | --- | --- | --- |
| Screening | X |  |  | X |
| Consent and Enrollment | X |  |  | X |
| Questionnaires | X |  |  | X |
| Vestibular/Ocular Motor Screening (VOMS) | X |  |  | X |
| 2-Minute Walk | X |  |  | X |
| Clinical Turns Course (CTC) | X |  |  | X |
| Dix-Hallpike | X |  |  | X |
| Video Head Impulse Test (vHIT) | X |  |  | X |
| Dynamic Visual Acuity (DVA) | X |  |  | X |
| 6-week Rehabilitation |  |  | X |  |
| 7-Days Home Monitoring |  | X |  |  |
| Patient Global Impression of Change (PGIC) |  |  |  | X |
| Total time | 5 hours | 70 hours | 720 minutes | 4 hours |

**RISKS AND DISCOMFORTS:**

Loss of confidentiality: The computer that will store the project database will be protected by current network security. An institutional computer account and password will be required to access the computer storing the recruitment database and the images. Data will be maintained on secure, encrypted servers maintained by OHSU.

Loss of privacy: You will be assigned a coded identification number and this number will be used to keep track of your data. Only individuals who are specifically authorized to view private health information by the principal investigator will have access to information that could be used to identify you. Thus, the risk to you in this research is in the categories of unlikely physical risks and potential loss of information privacy.

Musculoskeletal injury: There is a low risk of joint, tendon, or muscle pain, inflammation, or swelling during or after the gait and balance testing session. This risk is reduced by the mild nature of the gait and balance testing administered by well-trained research assistants.

Exacerbation of symptoms: There is a low risk that your symptoms such as dizziness, headache or nausea will become worse by performing the tests and by doing physical therapy. We will try to monitor your symptoms and provide breaks.

Emotional Distress: Some of the questions asked may be personal or embarrassing. You have the right to refuse to answer any of the questions if you do not want to. Additionally, you may learn information about your balance and walking and that could be upsetting to you.

Falls: The balance and walking tasks may cause you to lose your balance or fall. However, our trained research assistants will walk alongside you at all times; if you happen to lose your balance, our research assistant will be there to assist and prevent a fall. All safety measures will be taken to ensure a secure and comfortable environment. You will be allowed to take breaks during the balance and walking tasks whenever necessary. It is unlikely that you will fall.

Wearable sensor system: The wearable sensors worn on the body are commonly use in other laboratory studies assessing balance and gait. There is no identifiable information stored on these sensors. These sensors are non-invasive but may cause minimal discomfort similar to wearing a headband, wristwatch or belt.

Biofeedback sensor system: These sensors used for biofeedback are the same as the sensors in the wearable sensor system. There is no identifiable information stored on theses sensors. These sensors are non-invasive and may cause minimal discomfort similar to wearing a headband, wristwatch or belt.

**BENEFITS:** You may or may not personally benefit from being in this study. However, by serving as a subject, you may help us learn how to benefit patients in the future.

**ALTERNATIVES:** You may choose not to participate in this study.

**CONFIDENTIALITY AND PRIVACY OF YOUR PROTECTED HEALTH INFORMATION:**

We will not use your name or your identity for publication or publicity purposes. If you sign this form, you are agreeing that OHSU may use and disclose protected health information collected and created in this research study. We will be accessing information from your medical chart that was collected in your medical examination regarding your mTBI, if applicable.

The specific health information that we collect may include:

1. Health information about your mTBI
2. Body motion
3. Responses to questionnaires
4. Video recordings of the balance and gait tests

The purpose of this information is:

1. To learn more about the condition being studied
2. To improve health care for persons with the condition being studied
3. To analyze research results
4. To complete research obligations in this study
5. For teaching purposes

The persons who are authorized to use and disclose this information are: all investigators and co-investigators who are participating in the conduct of this research protocol, the Regional Health Command Central, Institutional Review Boards, and the Army Human Research Protection Office. The Department of Defense is authorized to access research records as part of its human subject’s protection oversight activities. Data will be shared for research purposes through the Federal Interagency Traumatic Brain Injury Research Informatics System (FITBIR). All links with your identity will be removed from the data before they are shared. Only de-identified data, which does not include anything that might directly identify you, will be shared with FITBIR users and the general scientific community for research purposes.

The persons who are authorized to receive this information are: the sponsors of the study and the Office for Human Research Protections.

We may continue to use and disclose protected health information that we collect from you in this study indefinitely*.*

While this study is still in progress, you may not be given access to medical information about you that is related to the study. After the study is completed and the results have been analyzed, you will be permitted access to any medical information collected about you in the study.

You have the right to revoke this authorization and can withdraw your permission for us to use your information for this research by sending a written request to the principal investigator listed on page one of the consent and authorization form. If you do send a letter to the principal investigator, the use and disclosure of your protected health information will stop as of the date she receives your request. However, the principal investigator is allowed to use information collected before the date of the letter or collected in good faith before your letter arrives. Revoking this authorization will not affect your health care or your relationship with OHSU. The information about you that is used or disclosed in this study may be re-disclosed and no longer protected under federal law.

**COSTS:** There is no cost to you to be in this study.

You will receive $75 for the first testing session and $100 for post intervention assessment. If you are asked to wear the sensors at home for 7 days, you will receive an additional $10/day totaling $70.

**LIABILITY:** If you believe you have been injured or harmed while participating in this research and require immediate treatment, contact Laurie King, Ph.D., P.T., MCR (503) 418-2602.

You have not waived your legal rights by signing this form. If you are harmed by the study procedures, you will be treated. OHSU does not offer to pay for the cost of the treatment. Any claim you make against OHSU may be limited by Oregon Tort Claims Act (ORS 30.260 through 30.300). If you have questions on this subject, please call the OHSU Research Integrity Office at (503) 494-7887.

This federally funded study does not have the ability to provide compensation for research-related injury. If you are injured or become ill from taking part in this study, it is important to tell your study doctor. Emergency treatment may be available but you or your insurance company will be charged for this treatment.

**PARTICIPATION:** If you have any questions regarding your rights as a research subject, you may contact the OHSU Research Integrity Office at (503) 494-7887. You do not have to join this or any research study. If you do join, and later change your mind, you may quit at any time. If you refuse to join or withdraw early from the study, there will be no penalty or loss of any benefits to which you are otherwise entitled.

Your health care provider may be one of the investigators of this research study, and as an investigator is interested in both your clinical welfare and in the conduct of this study. Before entering this study or at any time during the research, you may ask for a second opinion about your care from another doctor who is in no way involved in this project. You do not have to be in any research study offered by your physician.

**Statistical Analysis Plan for Wearable Technology to Characterize and Treat mTBI Subtypes: Biofeedback-Based Precision Rehabilitation (SuBTyPE)**

***Aim 1***

The *primary outcome* for Aim 1 is the Patient Global Impression of Change (PGIC). It is an ordinal variable where participants rate their global assessment of change on a scale from 1 to 7. To test whether the group with multidimensional real-time biofeedback has larger improvements in the PGIC than the group with standard care physical therapy, we will first conduct a Mann-Whitney U test to assess group differences and a Chi-square test to compare responders vs. non-responders in PGIC scores between groups. Responsiveness to rehabilitation will be determined by the participants' score on the PGIC after rehabilitation. PGIC score will be dichotomized by “responder” or “non-responder”. We will classify participants who score 6 or above on the PGIC as “responders” and those who score 5 or below as “non-responders”. Then we will perform subsequent analyses using ordinal logistic regression to examine group differences accounting for covariate effects (e.g., age, gender, PTSD, etc.). Additionally, we will also conduct a logistic regression on responder vs. non-responder as the binary outcome to assess differences between groups accounting for covariates.

*Secondary outcomes* (e.g., patient-reported symptoms, clinical and instrumented objective assessments) will be measured at baseline and post-rehabilitation. All secondary outcomes will be assessed for normality and we will perform a data transformation (e.g., log transformation) when appropriate. We will calculate the pre-post change in each secondary outcome for each participant. To test the difference in pre-post change between the multidimensional real-time biofeedback group and the standard care group, we will conduct a two-sample t-test or a Mann-Whitney U test for each secondary outcome. We will also employ mixed effects models to assess the differences in change in secondary outcomes between the real-time biofeedback and standard care group. Two fixed effects will be included in the model: 1) group effect (real-time biofeedback vs. standard care) and 2) time effect (baseline and post-rehabilitation). The interaction between group and time (group x time) will also be included. We will include covariates in the fully adjusted model. Random intercepts will be included to account for the clustering effect within subjects over time. We will use an inverse probability weighting (IPW) approach to account for participant attrition in the study and perform sensitivity analyses for IPW. We will also compare the descriptive information on demographic and clinical variables between those who drop out of the study and those who complete the study.

***Aim 2***

In this exploratory aim, we will: 1) assess high vestibular/ocular (V/O HI) versus low vestibular/ocular (V/O LO) participants’ responsiveness to rehabilitation and 2) explore the relationship between subjective and objective measures of V/O domains. First, we will examine the distribution of PGIC scores and conduct a Mann Whitney U test of PGIC scores between V/O high and V/O low groups. Then we will perform a Chi-square test to compare responders vs. non-responders (based on PGIC scores) between Hi V/O and LO V/O groups regardless of randomization group assignment. Then we will assess the PGIC as an ordinal outcome variable and test group differences (V/O HI vs. V/O LO) in responsiveness using ordinal logistic regression. Additionally, we will conduct a logistic regression on responder vs. non-responder as the binary outcome to assess differences between V/O HI group and V/O LO group. Both regression models will allow us to control for covariates (e.g., age, gender, days since injury, intervention arm) in the models. We will use the data to obtain estimates to inform future studies and power calculations. Finally, to explore the strength of associations between the Patient-reported, Clinical, and Instrumented V/O measures, we will measure the associations using Pearson correlation coefficients for continuous variables and Spearman correlation coefficients for ordinal variables.

***Aim 3***

Participants will be grouped according to their level of severity of V/O deficits (HI or LO V/O deficits), regardless of intervention group. Prior to detailed statistical modeling, we will compute quality metrics of daily life mobility quantified from instrumented socks summarized over the duration the devices were worn by each participant (for those that wore the devices for approximately 7 days and approximately 10 hours/day). Specifically, we will provide both statistical and graphical evaluation for the distributions of turn quality metrics: (Turn ngle and its variability (primary), Turn duration and its variability, , and Turn peak velocity and its variability), and then transform the values if necessary to better meet model assumptions. We will also examine quantitative activity measures including Activity rate, Number of steps per day and Number of turns per hour). We will examine the distribution of the metrics in each group. The descriptive statistics of metrics for each group will be reported as mean (SD). To test whether daily life mobility measures differ between V/O HI and V/O LO groups, we will test the metrics for group differences using a two sample t-test. In addition, we will conduct subsequent analyses to compare metrics between two groups accounting for covariates using the multivariate generalized linear modeling (GLM) approach. The model will be constructed for each metric (outcome) separately. The group estimates will be assessed with and without the co-existing covariates to explore potential confounding factors, which allows us to test for group differences while controlling for the possible effects of other covariates. In addition, we will explore other quality and quantity metrics and and will also examine the distributions using the same method and conduct two independent sample t-test between two groups for the completeness of the analysis.

To summarize the healthy military normative group, we will similarly collect data from instrumented socks worn over an approximate 7-day period. We will examine the distribution of the metrics and provide descriptive statistics including means, standard deviations, median and 25^th^, 75^th^ percentiles. Additionally, we will check the normality of the data. This will provide preliminary data for our next studies exploring daily life mobility in those cleared to return to duty after mTBI.
